# α-Synuclein Aggregates Increase the Conductance of Substantia Nigra Dopamine Neurons, an Effect Partly Reversed by the KATP Channel Inhibitor Glibenclamide

**DOI:** 10.1523/ENEURO.0330-20.2020

**Published:** 2021-01-13

**Authors:** E. Hill, R. Gowers, M. J. E. Richardson, M. J. Wall

**Affiliations:** 1 School of Life Sciences, University of Warwick, Coventry CV4 7AL, United Kingdoms; 2 Warwick Mathematics Institute, University of Warwick, Coventry CV4 7AL, United Kingdom; 3 Institute for Theoretical Biology, Department of Biology, Humboldt-Universität zu Berlin, Berlin 10115, Germany

**Keywords:** α-synuclein, dopamine, electrophysiology, KATP, patch clamp, substantia nigra

## Abstract

Dopaminergic neurons (DNs) in the substantia nigra pars compacta (SNpc) form an important part of the basal ganglia circuitry, playing key roles in movement initiation and coordination. A hallmark of Parkinson’s disease (PD) is the degeneration of these SNpc DNs leading to akinesia, bradykinesia and tremor. There is gathering evidence that oligomeric α-synuclein (α-syn) is one of the major pathologic species in PD, with its deposition in Lewy bodies (LBs) closely correlated with disease progression. However, the precise mechanisms underlying the effects of oligomeric α-syn on DN function have yet to be fully defined. Here, we have combined electrophysiological recording and detailed analysis to characterize the time-dependent effects of α-syn aggregates (consisting of oligomers and possibly small fibrils) on the properties of SNpc DNs. The introduction of α-syn aggregates into single DNs via the patch electrode significantly reduced both the input resistance and the firing rate without changing the membrane potential. These effects occurred after 8–16 min of dialysis but did not occur with the monomeric form of α-syn. The effects of α-syn aggregates could be significantly reduced by preincubation with the ATP-sensitive K^+^ channel (KATP) inhibitor glibenclamide. These data suggest that accumulation of α-syn aggregates in DNs may chronically activate KATP channels leading to a significant loss of excitability and dopamine release.

## Visual Abstract

**Figure:**
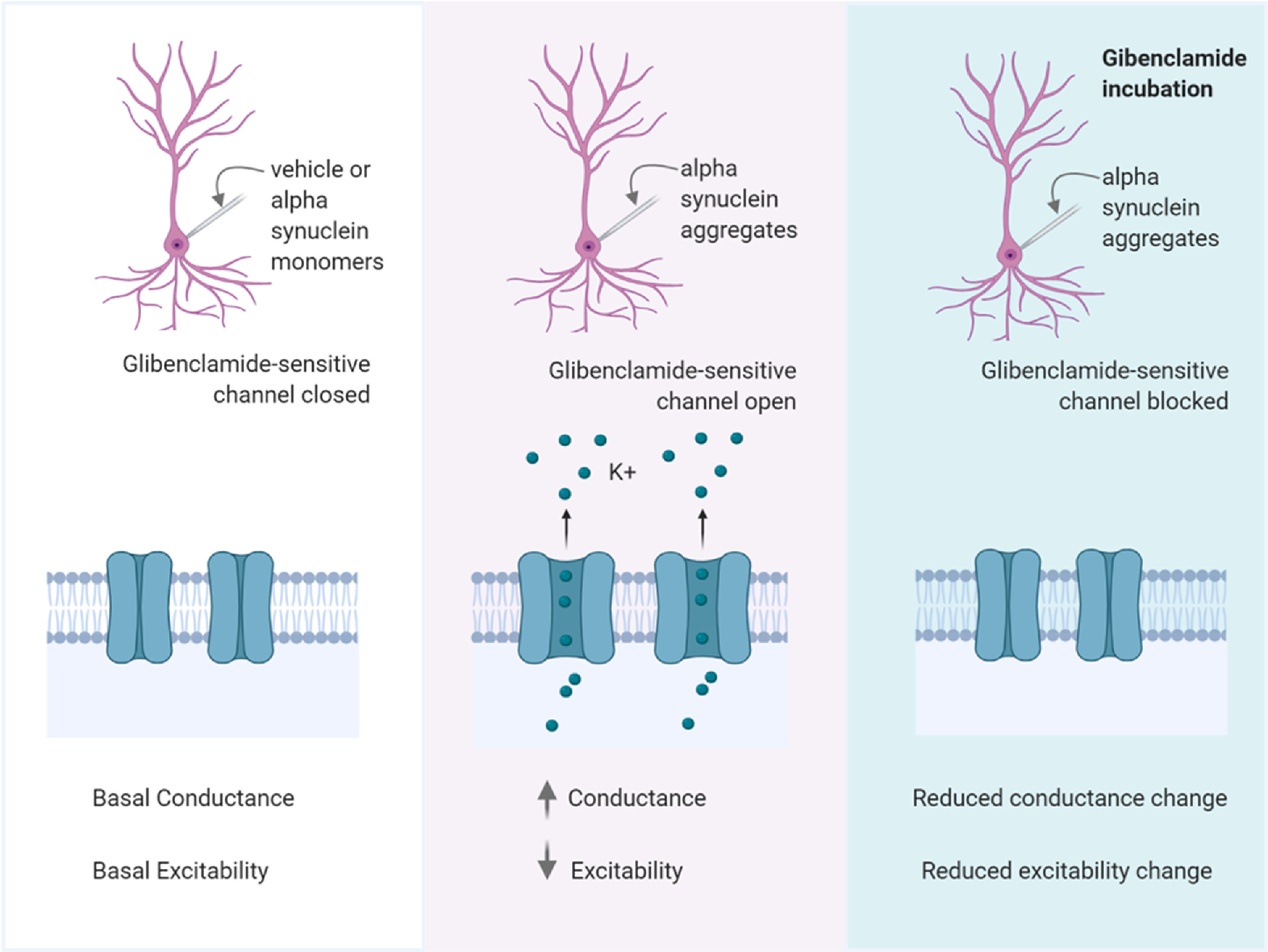

## Significance Statement

α-Synuclein (α-syn) oligomers are one of the key toxic species in Parkinson’s disease (PD), with their accumulation leading to dopamine neuron dysfunction. Introducing α-syn aggregates (oligomers and possibly small fibrils) into single substantia nigra dopamine neurons led to a marked increase in whole-cell conductance and a corresponding fall in the firing rate. These changes were diminished by inhibiting ATP-sensitive K^+^ channels (KATPs). Thus, the build-up of α-syn oligomers during the progression of PD could chronically shunt dopamine neurons, via channel activation (which may include KATP channel activation) to reduce dopamine release.

## Introduction

Mid-brain dopaminergic neurons (DNs) play major roles in the control of movement, emotion, arousal and reward behavior. They possess large numbers of projections, have pacemaker activity (firing action potentials at rest) and are therefore highly energy intensive. This makes them particularly sensitive to oxidative damage (which can lead to mitochondrial dysfunction, Michel et al., 2016), and they are the most susceptible to degeneration in Parkinson’s disease (PD; Damier et al., 1999). DNs are lost over the progression of PD, primarily from the substantia nigra pars compacta (SNpc) but later in the disease there is also loss from the ventral tegmental area (VTA). Symptoms of PD only become apparent when 70–80% of the dopamine input to the striatum is lost (Bernheimer et al., 1973) making it difficult to detect PD at early stages. Understanding what happens to these DNs early in pathology is important as it could provide improved diagnosis and potentially new targets to prevent or slow disease progression.

α-Synuclein (α-syn) is a small, native intracellular protein, found primarily at presynaptic terminals (Iwai et al., 1995) where it contributes to neurotransmitter uptake and vesicle recycling. It can also localize to the mitochondria and the nucleus (Burré et al., 2010). Molecules of α-syn are intrinsically disordered (Alderson and Markley, 2013) but can become ordered on aggregation, triggering a pathologic cascade. First α-syn aggregates into soluble oligomers (dimers and trimers) and then it can go on to form longer fibrils and ultimately it can form insoluble Lewy bodies (LBs; Spillantini et al., 1997). LBs are a key marker of pathology in the brains of PD patients with their abundance correlating with disease severity. LB formation involves a complex interplay between α-syn fibrillization, posttranslational modifications, and interactions of α-syn aggregates with membranous organelles. LB formation can lead to the disruption of mitochondrial function, to synapse dysfunction and of a decline in general cell function (Mahul-Mellier et al., 2020). However, there is also evidence that the small soluble oligomers contribute to toxicity (Winner et al., 2011; for review, see Bengoa-Vergniory et al., 2017). At the presynaptic terminal, oligomeric α-syn can induce synaptic dysfunction by disrupting vesicle trafficking (Jakes et al., 1994). It also interferes with synaptobrevin in the SNARE complex which is vital for the fusion of vesicles with the plasma membrane for neurotransmitter release (Murphy et al., 2000; Burré et al., 2010; Lashuel et al., 2013). Kaufmann et al. (2016) showed that α-syn oligomers reduced the excitability and input resistance of cortical pyramidal cells.

In this study, we have used whole-cell patch-clamp recording to provide a detailed characterization of the electrophysiological effects of introducing aggregated α-syn (oligomers plus a small proportion of fibrils) into single DNs in the substantia nigra of mouse brain slices. Since the α-syn aggregates are only introduced into one neuron, there will be no compensation within the circuit and any slow cellular uptake steps (which may occur if α-syn aggregates were applied extracellularly) are removed. Each cell acts as its own internal control, as at the time of whole-cell breakthrough the α-syn aggregates will not have diffused into the neuron. This approach has successfully been used to examine the effects of oligomeric tau on pyramidal neurons (Hill et al., 2019) and the effects of α-syn oligomers on Layer V pyramidal cells in the neocortex (Kaufmann et al., 2016).

As well as using standard current–voltage (SIV) relationships (with step-current injections) to measure the changes in DN electrophysiology, we have also utilized the dynamic IV (DIV) protocol. This was originally applied with cortical pyramidal neurons and interneurons (Badel et al., 2008a,b; Harrison et al., 2015) and can be used to develop simplified, but empirically verified quantitative models of their responses. Although the cortical cells in the original study exhibit nonlinear voltage and calcium-activated currents, under ongoing *in vivo*-like fluctuating current stimulation, their response could be well captured by an effective linear IV curve (away from action potential threshold). DNs, however, have more strongly expressed non-linearities (Richards et al., 1997; Neuhoff et al., 2002), and it can therefore be anticipated that the resulting DIV curves will diverge from an Ohmic linear form below threshold. Despite this, we have determined that the DIV can still be used to accurately extract a number of key electrophysiological parameters.

Using these approaches, we have characterized the real-time effects of α-syn aggregates on the electrophysiological properties of single neurons. We find that their major effect is to induce an increase in whole-cell conductance, which significantly dampens neuronal excitability and firing rate. These effects were partially but significantly reduced by glibenclamide, an ATP-sensitive K^+^ channel (KATP) inhibitor, suggesting a role for these channels in the pathologic actions of α-syn aggregates.

## Materials and Methods

### Preparation of acute brain slices

All experiments were approved by the local Animals Welfare and Ethics Board (AWERB) at the University of Warwick. C57/Bl6 mice (two to three weeks; of either sex) were killed by cervical dislocation and decapitated in accordance with the United Kingdom Animals (Scientific Procedures) Act (1986). The brain was rapidly dissected and kept on ice. The cerebellum was removed, and the rostral section of the brain was trimmed. The brain was then mounted rostral side down. Coronal slices (350 μm) were cut with a Microm HM 650V microslicer in cold (2–4°C) high Mg^2+^, low Ca^2+^ aCSF, composed of the following: 127 mm NaCl, 1.9 mm KCl, 8 mm MgCl_2_, 0.5 mm CaCl_2_, 1.2 mm KH_2_PO_4_, 26 mm NaHCO_3_, and 10 mm D-glucose (pH 7.4 when bubbled with 95% O_2_ and 5% CO_2_, 300 mOsm). Slices were stored at 34°C in standard aCSF (1 mm Mg^2+^ and 2 mm Ca^2+^) for at least 1 h before recording and were viable for up to 8 h.

### Preparation of α-syn aggregates

Recombinant human α-syn protein aggregates were purchased from Abcam (ab218819). These aggregates are advertised as preformed fibrils (PFFs) which was confirmed using negative-stain electron microscopy. Since we wanted to introduce smaller oligomeric species of α-syn into neurons, the PFFs were first broken down into smaller aggregates before recordings were made. PFF samples were sonicated (as in Polinski et al., 2018) for 15 min (50–60 Hz) at room temperature using a Grant Ultrasonic XUBA1 bath. Recombinant human α-syn protein in monomeric form (ab218818) was used as a control and negative-stain electron microscopy was used to confirm it was not aggregated.

### Transmission electron microscopy (TEM)

Formvar/carbon-coated 300-mesh copper grids (#S162, Agar Scientific) were glow-discharged using the ELMO system from Cordouan Technologies. Five microliters of α-syn species (monomer, PFF, or sonicated PFFs) were pipetted onto the grid and allowed to bind for 1 min. Excess samples were removed with a strip of filter paper, and 5 μl of 2% uranyl acetate was added for 1 min. After removing the excess stain with a strip of filter paper, the grids were imaged using a JEOL-2100F TEM.

### Whole-cell patch-clamp recording

A slice was transferred to the recording chamber, submerged and perfused (2–3 ml min^−1^) with aCSF at 30–32°C. Slices were visualized using IR-DIC optics with an Olympus BX151W microscope (Scientifica) and a CCD camera (Hitachi). Whole-cell current-clamp recordings were made from DNs in the SNpc using patch pipettes (5–10 MΩ) manufactured from thick-walled glass (Harvard Apparatus). Intracellular solution contained the following: 135 mm potassium gluconate, 7 mm NaCl, 10 mm HEPES, 0.5 mm EGTA, 10 mm phosphocreatine, 2 mm MgATP, and 0.3 mm NaGTP (293 mOsm, pH 7.2). The intracellular solution was filtered (0.2 μm) before the addition of α-syn aggregates (2 μl of a 69 μm stock into 275-μl intracellular solution to give final concentration of 500 nm) as filtering reduces the aggregate concentration (Kaufmann et al., 2016; Hill et al., 2019). The molar concentrations of α-syn species are based on the molar mass of monomeric α-syn (14 kDa) because of the likelihood that samples will contain a range of aggregate sizes. This method has been used in similar studies (see Hill et al., 2019; Thakur et al., 2019) and will result in an overestimate of aggregate concentration. For example, if aggregates are on average tetramers, then the concentration of aggregates will be 4× lower than reported. A subset of neurons was filled with AF594 dye (50 μm) via the patch pipette for immunohistochemistry. Voltage recordings were made using an Axon Multiclamp 700B amplifier (Molecular Devices) and digitized at 20 kHz. Data acquisition and analysis were performed using pClamp 10 (Molecular Devices). Recordings from neurons that had a resting membrane potential of between −55 and – 75 mV at whole-cell breakthrough were accepted for analysis. The bridge balance was monitored throughout the experiments and any recordings where it changed by >20% were discarded.

### Stimulation protocols

#### SIV protocol

SIVs were constructed by injecting step currents (3 s duration, with a 5 s interval) starting at −200 pA and then incrementing by either 50 or 100 pA until a regular firing pattern was induced.

#### Naturalistic current injection

A naturalistic, fluctuating current (40 s in duration; for details, see below) was injected into neurons, and the resulting voltage trace was used to measure the frequency of action-potential firing (using threshold detection in Clampfit).

### Immunohistochemistry

After completing the electrophysiology recordings from DNs, with the addition of AF594 dye (50 μm) in the patch pipette, slices (350 μm) were fixed in 4% PFA for 45 min at room temperature and then overnight at 4°C. The tissue was then washed five times for 5 min with PBS. The slices were then blocked for 1 h (1% BSA, 0.4% Triton X-100 in PBS, 400 μl per slice), then washed three times for 5 min with PBS. The primary antibodies against tyrosine hydroxylase (1:1000, sheep), was added to the slices (250 μl per slice) for 1 h at room temperature and then kept at 4–8°C overnight. Slices were washed five times for 5 min with PBS and then the secondary antibody was added (anti-sheep 488, 1:500, 200 μl per slice) for 4 h at room temperature. The slices were then washed five times for 5 min with PBS, and then mounted on glass slides with Vectashield (Vector Laboratories). All imaging was carried using confocal microscopy (Leica 710 and Zen Black for image acquisition and processing). Controls were conducted without incubating with the primary antibodies and showed no fluorescence.

### Drugs and substances

Recombinant human α-syn aggregates were purchased from Abcam (ab218819) along with the corresponding monomers (ab218818). Dopamine (HB1835) and ZD 7288 (HB1152) were purchased from Hello Bio (HB1835). Glibenclamide (PHR1287) was purchased from Sigma-Aldrich. The sheep polyclonal anti-tyrosine hydroxylase was purchased from Merck (AB1542) and donkey anti-sheep 488 secondary from Invitrogen (A11015). The Alexa Fluor 594 hydrazide dye was purchased from Invitrogen (10072752).

### Statistics

Each recorded cell is one data point. All experimental conditions were measured using multiple animals as only one cell was recorded per slice and recording conditions were interleaved to remove bias introduced from individual animals. Data points for each experimental condition were derived from a minimum of four individual animals (the exact numbers are provided in the figure legends). Statistical analysis was performed using non parametric methods: Kruskal–Wallis one-way ANOVA, Mann–Whitney, and Wilcoxon signed-rank tests as required (full details provided in Table 2). All data are represented as mean and SEM with individual experiments represented by single data points. SDs are given in Table 1.

**Table 1:** Electrophysiological parameters measured for DNs at time 0 for all experimental treatments

	Vehicle/control			α-Syn aggregates			α-Syn monomers			Glib + vehicle			Glib + α-syn aggregates		
Parameter	Mean	SEM	SD	Mean	SEM	SD	Mean	SEM	SD	Mean	SEM	SD	Mean	SEM	SD
RMP (mV)	−55.8	±1.2	±3.79	−55.4	±1.23	±4.10	−56.8	±1.30	±3.18	−53.2	±2.14	±5.24	−60.4	±1.82	±4.08
R in (MΩ)	338	±17.6	±55.8	312	±17.1	±56.8	359	±42.2	±103.3	386	±25.2	±61.7	294	±41.9	±93.6
Firing rate (Hz)	1.63	±0.39	±0.12	2.51	±1.57	±0.47	2.38	±1.14	±0.46	2.27	±1.15	±0.47	2.04	±1.29	±0.52
C (pF)	88.8	±5.74	±14.1	107.8	±5.84	±14.3	80.73	±15.1	±37.0	81.16	±8.54	±20.9	117	±22.3	±54.7
g (nS)	2.29	±0.2	±0.49	2.78	±0.2	±0.49	1.99	±0.3	±0.73	2.39	±0.29	±0.71	3.67	±0.55	±1.35

**Table 2:** A table of all of the statistical tests and associated result

Data	Figure	Statistical test	Test values	*p* value
0 min, all conditions, RMP	4	Kruskal–Wallis ANOVA	*H*_(4)_ = 0.5385	*p* = 0.4657
0 min, all conditions, IR	4	Kruskal–Wallis ANOVA	*H*_(4)_ = 2.109	*p* = 0.2727
0 min all conditions, FR	4	Kruskal–Wallis ANOVA	*H*_(4)_ = 1.395	*p* = 0.8511
0 vs 32 min, α-syn aggregates, IR	4	Wilcoxon signed-rank test	W(+) = 2.0, W(–) = 64.0, *Z* = 2.7	*p* = 0.0029
0 vs 32 min, α-syn monomers, IR	4	Wilcoxon signed-rank test	W(+) = 6.5, W(–) = 14.5, *Z* = 0.73	*p* = 0.4688
0 vs 32 min, vehicle, IR	4	Wilcoxon signed-rank test	W(+) = 46.0, W(–) = 9.0, *Z* = −1.83	*p* = 0.0645
32 min, α-syn aggregates, α-syn monomers and vehicle, IR	4	Kruskal–Wallis ANOVA	*H*_(2)_ = 14.53	*p* = 0.0007
16 min, α-syn aggregates, α-syn monomers and vehicle, IR	4	Kruskal–Wallis ANOVA	*H*_(2)_ = 10.67	*p* = 0.0048
0 vs 32 min, vehicle, RMP	4	Wilcoxon signed-rank test	W(+) = 41.5, W(–) = 3.5, *Z* = −2.19	*p* = 0.0234
0 vs 32 min, α-syn aggregates, RMP	4	Wilcoxon signed-rank test	W(+) = 61.0, W(–) = 5.0, *Z* = −2.44	*p* = 0.0107
0 vs 32 min, α-syn monomers, RMP	4	Wilcoxon signed-rank test	W(+) = 19.5, W(–) = 1.5 *Z* = −1.79	*p* = 0.0938
32 min, α-syn aggregates, α-syn monomers and vehicle, RMP	4	Kruskal–Wallis ANOVA	*H*_(2)_ = 2.234	*p* = 0.3273
0 vs 32 min, vehicle, FR	4	Wilcoxon signed-rank test	W(+) = 14, W(–) = 41.0 *Z* = −1.32	*p* = 0.1895
0 vs 32 min, α-syn aggregates, FR	4	Wilcoxon signed-rank test	W(+) = 1.0, W(–) = 65.0 Z = 2.8	*p* = 0.0020
0 vs 32 min, α-syn monomers, FR	4	Wilcoxon signed-rank test	W(+) = 11, W(–) = 10.0 *Z* = 0	*p* > 0.9999
32 min, α-syn aggregates, α-syn monomers and vehicle, FR	4	Kruskal–Wallis ANOVA	*H*_(2)_ = 10.21	*p* = 0.0061
0 min, all conditions, capacitance	5	Kruskal–Wallis ANOVA	*H*_(4)_ = 6.269	*p* = 0.18
0 vs 32 min, α-syn aggregates, membrane conductance	5	Wilcoxon signed-rank test	W(+) = 66.0 , W(–) = 0 *Z* = −2.89	*p* = 0.001
0 vs 32 min, vehicle, membrane conductance	5	Wilcoxon signed-rank test	W(+) = 71.0 , W(–) = 7 *Z* = −2.47	*p* = 0.009
32 min, α-syn monomers and vehicle, membrane conductance	5	Mann–Whitney test	Vehicle median = 2.820, *n* = 12, α-syn monomers = 2.820, *n* = 6, *U* = 36	*p* > 0.9999
32 min, α-syn aggregates and vehicle, membrane conductance	5	Mann–Whitney test	α-Syn aggregates median = 6.450, *n* = 11, vehicle median = 2.820, *n* = 12, *U* = 11	*p* = 0.0003
0 min, vehicle and vehicle + glib, RMP	6	Mann–Whitney test	Vehicle media*n* = −56, *n* = 10, vehicle + glib median = −54, *n* = 6, *U* = 21.50	*p* = 0.3791
0 min, vehicle and vehicle + glib, IR	6	Mann–Whitney test	Vehicle median = 338.3, *n* = 10, vehicle + glib median = 393, *n* = 6, *U* = 18.50	*p* = 0.2294
0 min, vehicle and vehicle + glib, FR	6	Mann–Whitney test	Vehicle median = 69.5, *n* = 10, vehicle + glib median = 86.5, *n* = 6, *U* = 25.50	*p* = 0.6548
0 vs 32 min, vehicle + glib, IR	6	Wilcoxon signed-rank test	W(+) = 9.0, W(–) = 12.0 *Z* = 0.21	*p* = 0.8438
0 vs 32 min, vehicle + glib, FR	6	Wilcoxon signed-rank test	W(+) = 7.0, W(–) = 14.0 *Z* = 0.63	*p* = 0.5625
0 vs 32 min, α-syn + glib, IR	6	Wilcoxon signed-rank test	W(+) = 5.0, W(–) = 16.0 *Z* = 1.04	*p* = 0.25
0 vs 32 min, α-syn + glib, FR	6	Wilcoxon signed-rank test	W(+) = 7.0, W(–) = 14.0 *Z* = 0.63	*p* = 0.353
0 vs 32 min, α-syn + glib, membrane conductance	7	Wilcoxon signed-rank test	W(+) = 21.0, W(–) = 0 *Z* = 0.63	*p* = 0.0313
32 min, vehicle + glib and α-syn + glib, membrane conductance	7	Mann–Whitney test	Vehicle + glib median = 4.675, *n* = 6, α-syn + glib median = 3.33, *n* = 6, *U* = 11	*p* = 0.3095
Normalized conductance over time, all conditions Conductance effect Time effect α-Syn aggregates vs control α-Syn aggregates vs monomers α-Syn aggregates vs vehicle + glib α-Syn aggregates vs α-syn + glib	7	Kruskal–Wallis ANOVA Dunn’s *post hoc* analysis	*F*_(4,180)_ = 6.806 *F*_(4,180)_ = 11.01	*p* < 0.0001 *p* < 0.0001 *p* < 0.0001 *p* = 0.0003 *p* < 0.0001 *p* = 0.0045

### Neuronal parameter extraction

Both the SIV curve (response to constant current inputs) and DIV curve (Badel et al., 2008a,b) methods were used to extract estimates for the cellular conductance under the various pharmacological conditions. The voltage was modelled by the following the current-balance equation:

(1) $$C\frac{dV}{dt}+I_{ion}=I_{inj},$$

where *C* is the capacitance, *I_ion_* is the summed intrinsic current (including voltage-gated and calcium-gated currents), and *I_inj_* is the current injected via the electrode during the different protocols. All computational modeling and data-fitting was conducted using custom written code in the Julia language (Bezanson et al., 2017) ported from published MATLAB code (Harrison et al., 2015).

#### SIV curve

To calculate neuronal resistance, the maximum voltage deflection to the −100-pA current step (before the sag, Fig. 2*A*, arrow) was measured (peak neuronal resistance). The position for the voltage measurement was determined on the voltage traces at time 0 (where there was a clear peak), and then the same position was used for the rest of the voltage traces at subsequent time points.

#### DIV curve

As well as measuring the static properties of the neuron at rest it is also useful to measure the effective parameters when the neuronal voltage is subject to a fluctuating drive, as is the case *in vivo*. To this end, we employed the DIV curve protocol (Badel et al., 2008) in which a stochastic current *I_inj_* is injected into the cell to induce a fluctuating voltage response and triggering of action potentials (Fig. 2*B*). A detailed description of the protocol has been previously published (Badel et al., 2008) as well as the computer code required (Harrison et al., 2015). In brief, a noisy current trace *I_inj_* is generated computationally (from the sum of two Ornstein–Uhlenbeck Gaussian processes with time constants τ_fast_ = 3 ms and τ_slow_ = 10 ms to mimic AMPA and GABA_A_ receptor time courses) and injected into the cell with the resultant voltage measured. The voltage Equation 1 can be re-arranged for the ionic current to give:

(2) $$I_{ion}=I_{inj}-C\frac{dV}{dt},$$

where the injected current is known, and the derivative can be calculated from measured voltage (with the capacitance extracted using the method in Badel et al., 2008). The ionic current and voltage time courses can then be plotted against each other (Fig. 2*C*, red scattered points). Given the interaction of the gated ionic currents with the stochastic injected current, there is significant scatter in the relation. However, the ionic current can be averaged in voltage slices to provide the DIV curve (Fig. 2*C*, black solid line, *D*, expanded view). Once the DIV curve has been obtained the various features can be extracted such as the ohmic conductance, the linear component of the IV curve at more hyperpolarized subthreshold voltage, and the excess depolarization-activated current seen in these cells near threshold.

By using both DIV and SIV we use two independent methods that induce distinct voltage dynamics to extract neuronal parameters thereby increasing the robustness of the data. These approaches also examine the neurons under different conditions. For SIV, the parameters are extracted from neurons that are in a simple dynamical state. In contrast the fluctuations induced by the DIV protocol result in a voltage that rapidly explores a much larger range.

## Results

### Characterizing DNs in the substantia nigra

**Figure 1.**
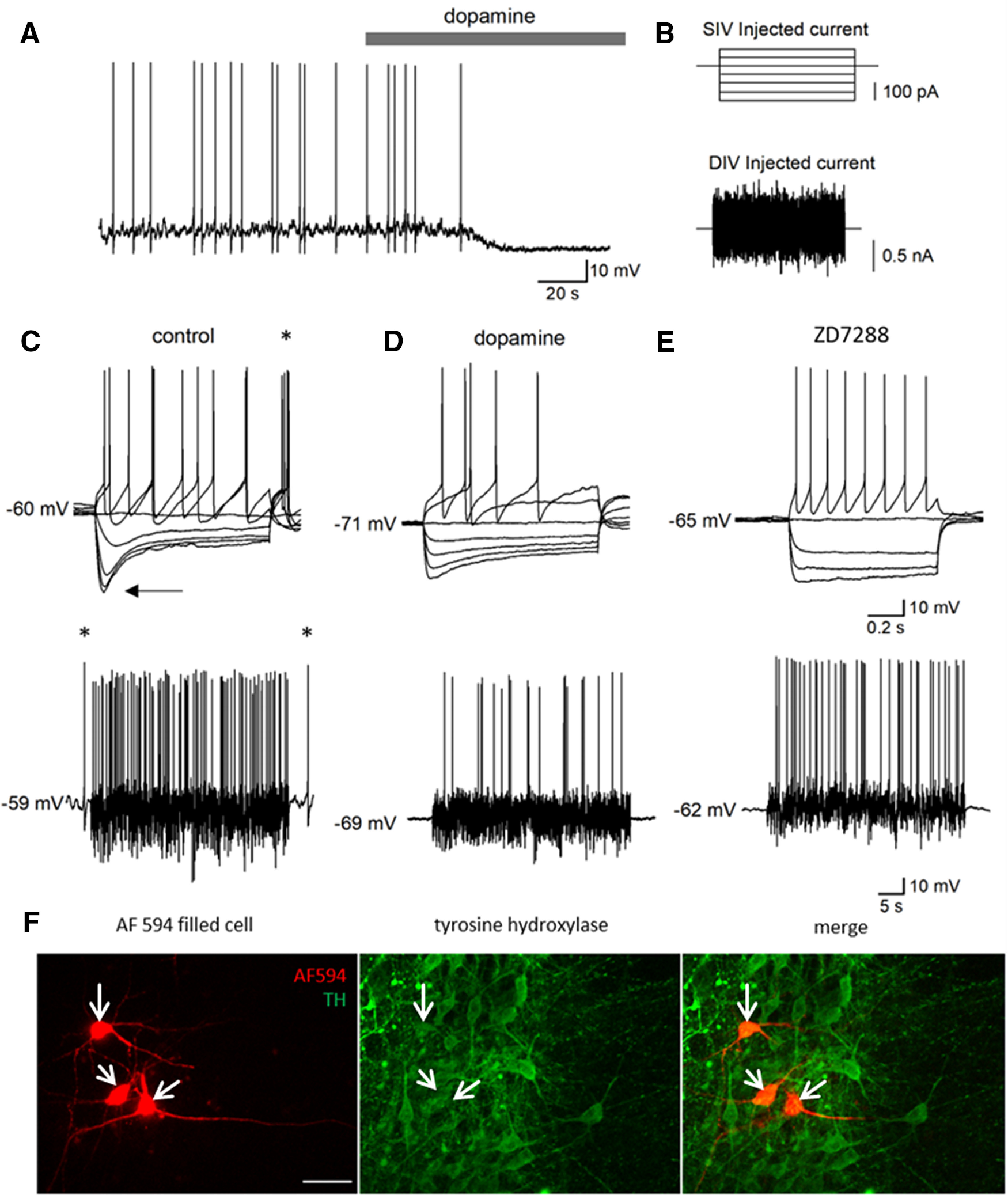
Whole-cell patch-clamp recording from DNs in the SNpc. ***A***, Example membrane potential trace recorded from a putative DN in the substantia nigra which showed characteristic spontaneous action potential firing. Dopamine (30 μm) hyperpolarized the neuron from −62 to −70 mV and stopped the action potential firing. ***B***, The different input currents delivered to putative DNs. SIV is the standard step current protocol, 3 s duration steps starting at −200 pA and increasing by 50 pA until the neuron exhibits a regular firing pattern. DIV uses a fluctuating naturalistic noisy current trace (for details, see Materials and Methods) which is also used to determine action potential firing rate. ***C***, top, Membrane potential traces in response to current steps. The recorded neuron displays characteristic features of DNs: a large sag in response to hyperpolarizing steps (arrow) and rebound firing (*). Bottom, Membrane potential trace from the same cell in response to naturalistic current injection. The neuron can be seen to be firing at rest (*) which is a characteristic feature of these neurons. ***D***, top, Membrane potential traces in response to current steps following application of dopamine (30 μm). The sag is reduced, the membrane potential hyperpolarized, the firing reduced, and the rebound firing is absent. Bottom, Membrane potential trace from the same cell in response to naturalistic current injection in dopamine (30 μm). The neuron has stopped firing at rest, fires less frequently during current application and is hyperpolarized. ***E***, top, Membrane potential traces in response to current steps in the presence of the I(h) blocker ZD7288 (100 μm) are similar to that previously reported for DN (Harris and Constanti, 1995). The sag response to hyperpolarizing current steps is markedly reduced, the resting membrane potential is hyperpolarized, the firing rate reduced and the voltage response following the spike is altered. Bottom, Membrane potential response of the same neuron to the naturalistic injected current in ZD7288 (100 μm). ***F***, Tyrosine hydroxylase (TH) immunohistochemistry confirms that the recorded neurons were DNs. Neurons were filled with AF594 dye (red) via the patch pipette and then slices were stained for TH (green). The merged image shows that the recorded neurons express TH and are therefore dopaminergic. Scale bar: 50 μm.

Whole-cell patch-clamp recordings were made from putative DNs located in the SNpc (identified by position in the slice). Neurons were initially confirmed to be SNpc DNs from their electrophysiological profile, which was consistent with previous studies (Grace and Onn, 1989; Richards et al., 1997; Krashia et al., 2017). Most of the recorded neurons displayed slow autonomous firing (36 out of 49 neurons, 73%, mean firing rate of 1.05 ± 0.082 Hz; Fig. 1*A*) consistent with the reported properties for dopamine neurons in the SNpc (Grace and Onn, 1989). These neurons were sensitive to dopamine (Lacey et al., 1989), with application of dopamine (30 μm) hyperpolarizing the membrane potential by 7.35 ± 1.24 mV and abolishing the spontaneous firing (Fig. 1*A*, *n *=* *8). The neurons showed a characteristic (Neuhoff et al., 2002) response to step-current injection (Fig. 1*B*), with a large sag in response to hyperpolarizing current steps (Fig. 1*C*) indicative of the presence of I(h). Following the termination of hyperpolarizing-current steps there was a rebound potential leading to firing in 33 out of 49 neurons (68%; Fig. 1*C*) another characteristic of I(h). Dopamine reduced the firing in response to the positive current steps and to the naturalistic current injection (firing rate reduced to 62.7 ± 0.2% of that in control). Dopamine also reduced the voltage response to hyperpolarizing current steps (indicative of an increase in whole-cell conductance) and also abolished the rebound firing (Fig. 1*D*). Application of ZD7288 (100 μm), a pharmacological inhibitor of I(h) in dopamine neurons (Harris and Constanti, 1995), greatly reduced the sag response produced by hyperpolarizing current steps and also abolished the rebound firing, confirming that they were I(h) mediated (Fig. 1*E*, *n *=* *6). Finally, to confirm that the neurons were dopaminergic, a subset of the recorded neurons (*n *=* *9) were filled with AF594 dye via the patch pipette and immunofluorescence staining was used to confirm that they were tyrosine hydroxylase-positive (Fig. 1*F*).

### Detailed analysis of the electrophysiology of DNs in the substantia nigra

**Figure 2.**
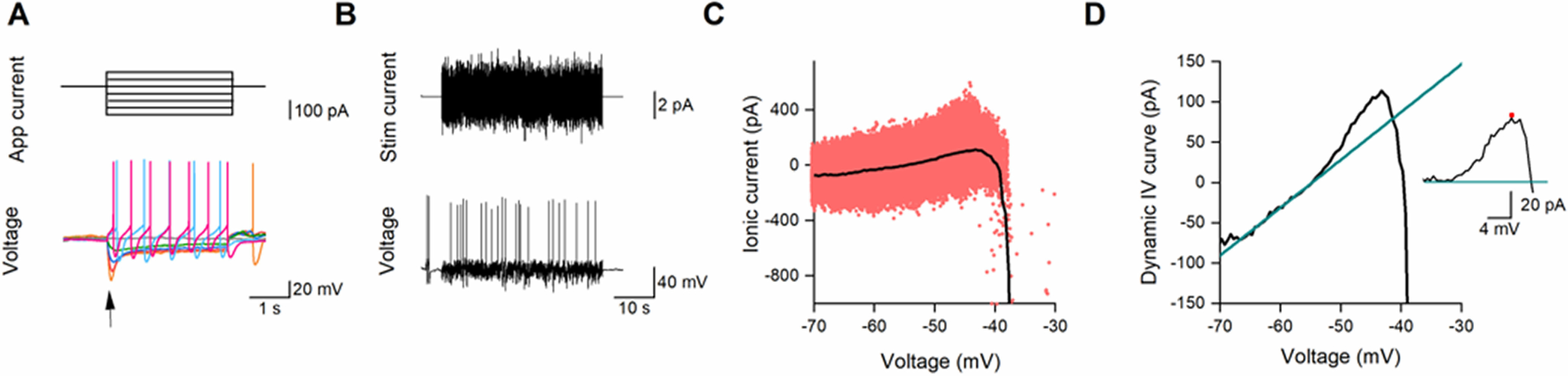
Extracting neuronal parameters using SIV (***A***) and DIV (***B–D***) methodologies. For the SIV measurements: (***A***) applied current and voltage response. Arrow shows location of voltage measurement for the calculation of neuronal resistance (peak voltage deflection). For the DIV measurements: (***B***) naturalistic stimulation current and voltage response. ***C***, The estimated ionic current as a scatter plot against voltage (points). The DIV curve (black) is the typical ionic current at a particular voltage where data within 200 ms postspike was not included to avoid the effects of transitory spike-generated currents. ***D***, The DIV curve (black) and its linear fit to the ohmic component (green, from hyperpolarized voltages to the resting voltage). Inset, Isolated outward current. For further details of the SIV and DIV fitting procedures, see Materials and Methods.

We used two approaches to characterize changes that occurred when SNpc DNs had either α-syn aggregates, monomers, or vehicle introduced. The first constituted of measurements from SIV curve protocols during which neurons received constant-current (step) inputs (Fig. 2*A*). The second approach measures neuronal properties using the DIV curve protocol (Badel et al., 2008a,b; Harrison et al., 2015), during which neurons were stimulated by a stochastic current mimicking fluctuating synaptic drive (Fig. 2*B–D*). This methodology allows for the average ionic current at a particular voltage to be measured at a good resolution over the full voltage range. The resulting DIV curve can be used to provide capacitance, ohmic conductance at hyperpolarized regimes as well as the strength of excess current seen near threshold in these cells (Fig. 2*B–D*). It is clear that the DIV curve diverges from linear at potentials above ∼−50 mV (but below action-potential threshold) which is characteristic of a depolarization-activated outward current (Fig. 2*D*). This effect of the outward current can be isolated by subtracting an extrapolated fit to the linear portion of the DIV curve (Fig. 2*D*, inset).

### Characterizing the structure of the α-syn aggregates

**Figure 3.**
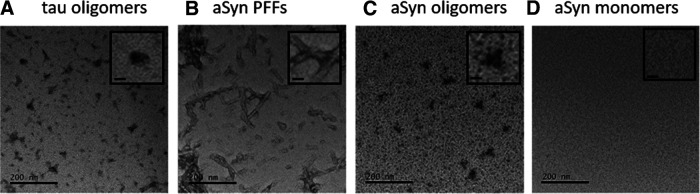
Structural analysis of α-syn samples. Electron micrographs of negative-stain-TEM transmission electron microscopy analyzed protein samples. The sample (5 μl) was applied onto a copper grid and fixed with uranyl acetate. Magnification is 60,000×. ***A***, Tau aggregates (for reference) showing aggregated form. Inset, Higher magnification showing annular form of aggregates. ***B***, α-Syn monomers show no aggregation. Inset, Higher magnification showing no structural form. ***C***, α-Syn aggregates display many large fibrils (PFFs). Inset, Example of fibrils at higher magnification. ***D***, α-Syn fibrils were added to intracellular patch solution and then sonicated for 15 min to form small aggregates. Inset, Annular form of aggregates. Scale bar: 200 nm, 20 nm .

Recombinant human α-syn protein was purchased from Abcam in the form of PFF aggregates (ab218819) and in the form of monomers (ab218818). Negative stain TEM was initially to confirm that the samples were either monomeric (Fig. 3*B*) or PFFs (Fig. 3*C*). To enable delivery of aggregates, mostly in oligomeric form, via the patch pipette, the PFFs were broken down. We used sonication (as in Polinski et al., 2018) for 15 min (50–60 Hz). We compared the structure of the α-syn aggregates (sonicated PFFs) to oligomeric tau which had an annular structure (Fig. 3*A*; Hill et al., 2019) to ensure that it consisted of oligomeric species (Fig. 3*B*). These oligomeric forms were stable for at least 3 h on ice. While the majority of the species in our samples had an oligomeric structure, we cannot exclude the possibility that other forms of aggregates exist (for example, small ∼50-nm fibrils; Polinski et al., 2018). We therefore use the term aggregates rather than oligomers. Monomeric or aggregated α-syn samples were introduced into DNs via the patch pipette.

### α-Syn aggregates but not monomers have marked effects on the electrophysiological properties of SN DNs

We introduced either aggregated or monomeric α-syn (500 nm, same concentration as Kaufmann et al., 2016) via the patch pipette during whole-cell current-clamp recording from identified SNpc DNs. To ensure consistency, all molar concentrations were based on the molar mass of monomeric α-syn as the samples probably contain a range of aggregate sizes (see above). Electrophysiological parameters were then measured from the voltage responses to step currents (SIV) and naturalistic current injections (DIV). The currents for SIV and DIV were injected at 8 min intervals for the duration of recordings (32 min; Kaufmann et al., 2016). In control experiments, the same volume of vehicle (2-μl PBS) was added to the intracellular solution. Recordings (α-syn aggregates, monomers, or vehicle) were made from SNpc DNs in interleaved slices to minimize variation.

**Figure 4.**
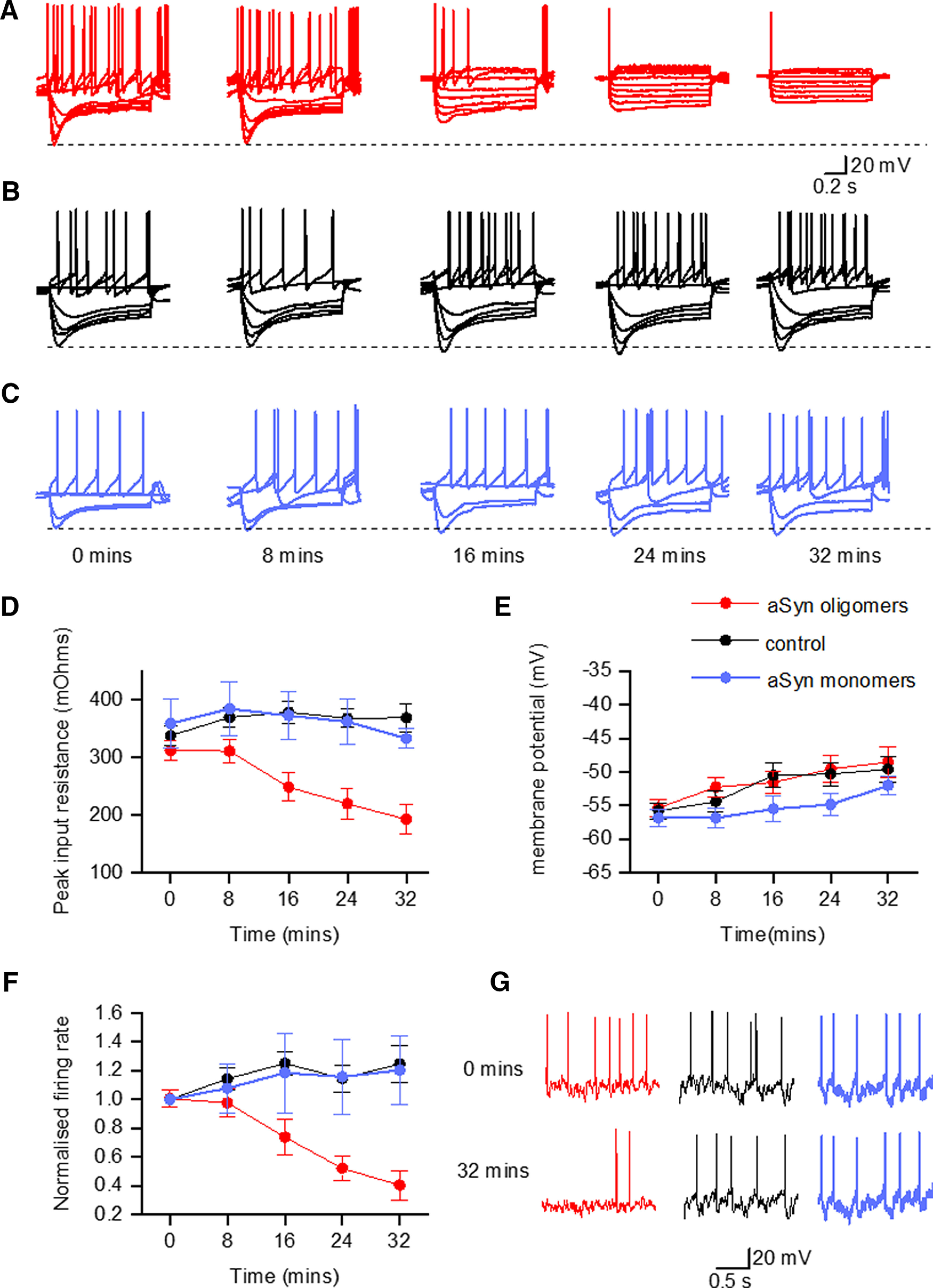
α-Syn aggregates induce a time-dependent decline in firing rate and significant fall in neuronal resistance. Example current–voltage relationships (SIV) for DNs injected with α-syn aggregates (***A***), vehicle (***B***), and α-syn monomers (***C***). Current steps (starting at –200 pA and increasing by 50 or 100 pA until a regular firing pattern was induced) were used to construct SIVs. Recordings display SIVs at time points between whole-cell breakthrough (0 min) and up to 32 min. The neuron which had the α-syn aggregates introduced (***A***) shows a clear reduction in the voltage responses and a fall in the firing rate at positive potentials, whereas the neurons that received either vehicle or α-syn monomers show no significant changes (***B***, ***C***). ***D***, Mean neuronal resistance plotted against time for control (vehicle) neurons and neurons that had either α-syn aggregates or monomers introduced. For the neurons injected with α-syn aggregates, after 32 min of recording, the resistance was significantly (*p* = 0.0029) reduced to 63 ± 9.21% of the resistance measured at 0 min. At the 32-min time point, there was a significant (*p* = 0.0002) difference in resistance between the neurons that received α-syn monomers or vehicle to those where α-syn aggregates were introduced. ***E***, Mean resting membrane potential plotted against time for control (vehicle) neurons and neurons that had either α-syn aggregates or monomers introduced. Under all experimental conditions, the neurons slowly depolarized over the time course of the recording. For vehicle, the mean depolarization (ΔVm) was 6.2 ± 1.82 mV after 32 min of recording (*p* = 0.0234), for α-syn monomers ΔVm was 3.8 ± 1.2 mV (*p* = 0.0938) and for α-syn aggregates ΔVm was 6.8 ± 1.85 mV (*p* = 0.0107). ***F***, Normalized firing rate plotted against time for control (vehicle) neurons and neurons that had either α-syn aggregates or monomers introduced (firing rate was measured from naturalistic current injection). Data are normalized to the firing rate at time 0 (whole-cell break through). Despite depolarizing by a comparable amount to the control (vehicle) and monomer introduced neurons, α-syn aggregates induced a significant reduction in the firing rate over time (*p* = 0.0020) consistent with the fall in input resistance. When comparing control (vehicle and α-syn monomers) and α-syn aggregates there was a significant difference in the firing rate at 32 min (*p* = 0.0061). ***G***, The same section of voltage trace taken during the injection of naturalistic current in example neurons injected with vehicle, α-syn monomers, and α-syn aggregates to illustrate the changes in firing pattern over the duration of recordings.

At time 0 (a few minutes following whole-cell breakthrough), there was no significant difference in the measured parameters from SIVs (membrane potential *p* = 0.4657, resistance *p* = 0.2747 and firing rate *p* = 0.8511; Table 1) between neurons which had received either vehicle, α-syn monomers or aggregates. Thus, the recording quality and neuronal properties were comparable between the experimental groups. However, as illustrated in the example in Figure 4*A*, there were marked changes in the SIV during recording from neurons in which α-syn aggregates had been introduced. The changes in SIV, reduction in voltage responses and decrease in firing rate at positive potentials, were indicative of a significant fall in neuronal resistance and the opening of a membrane channel. Such large changes in the SIV did not occur in the neurons that received either α-syn monomers or vehicle (Fig. 4*B*,*C*). For the neurons injected with α-syn aggregates, after 32 min of recording, the resistance was significantly (*p* = 0.0029, *n *=* *11) reduced to 63 ± 9.21% of the resistance measured at 0 min (Fig. 4*D*). There was no significant changes in the resistance for neurons injected with either α-syn monomers (*p* = 0.4688, *n *=* *6) or vehicle (*p* = 0.0645, *n *=* *10) over the duration of recordings (at 32 min input resistance (IR) was 94 ± 5% of the resistance measured at time 0 min for vehicle and 99 ± 9% of resistance measured at time 0 min for monomers; Fig. 4*D*). At the 32-min time point there was a significant (*p* = 0.0007) difference in resistance between the neurons that received α-syn monomers or vehicle to those where α-syn aggregates were introduced. We investigated the time at which α-syn aggregates began to affect neuronal resistance and found that the difference in resistance between control and aggregated and monomeric α-syn neurons first became significant at the 16-min time point (*p* = 0.0048; Fig. 4*D*). Thus, the resistance started to fall (presumably as membrane channels opened) between 8 and 16 min after α-syn aggregate introduction into the neuron at whole-cell break through.

The recorded neurons had a membrane potential of around −55 mV (see Table 1) at the start of recordings. For all experimental conditions, the membrane potential slowly and weakly depolarized over the duration of the recordings (Fig. 4*E*). For vehicle, the mean depolarization (ΔVm) was 6.2 ± 1.82 mV after 32 min of recording (*p* = 0.0234, *n *=* *10), for α-syn monomers ΔVm was 3.8 ± 1.2 mV (*p* = 0.0938, *n *=* *6), and for α-syn aggregates ΔVm was 6.8 ± 1.85 mV (*p* = 0.0107, *n *=* *11). There was no statistically significant difference between the membrane potentials of the control (vehicle and monomers) versus α-syn aggregates across the duration of the recordings (*p* = 0.3273). Thus, although α-syn aggregates markedly reduced neuronal resistance they did not produce a significant change in the membrane potential.

For control neurons (vehicle), although the membrane potential was depolarized, the change in firing rate did not reach significance (*p* = 0.1895, measured from the naturalistic current injection, firing rate at 32 min was 124.7 ± 1.3% of the firing rate at time 0 min, *n *=* *10; Fig. 4*F*,*G*). For neurons which received α-syn monomers, although the membrane potential was depolarized there was also no significant (*p* > 0.9999) change in the firing rate (measured from the naturalistic current injection, firing rate at 32 min was 120.4 ± 2.4% of the firing rate at time 0 min, *n *=* *6; Fig. 4*F*,*G*). For the neurons which had α-syn aggregates introduced, there was a significant (*p* = 0.0020) reduction in the firing rate (at 32 min the firing rate was 42.3 ± 9.3% of the firing rate measured at 0 min, *n *=* *11; Fig. 4*F*,*G*). When comparing control (vehicle and monomers) versus α-syn aggregates there was a significant difference in the firing rate at 32 min (*p* = 0.0061). This fall in firing rate induced by α-syn aggregates is consistent with the marked fall in neuronal resistance.

α-Syn aggregates decreased the occurrence of rebound firing following the termination of hyperpolarization steps [73% of neurons (8/11) showed rebound firing at 0 min, but only 18% (2/11) till showed it after 32 min of recording]. In contrast, all the control neurons that initially displayed rebound firing [60% (6/10)] still showed it after 32 min of recording. Aggregated α-syn also reduced the occurrence of tonic firing [64% of neurons (7/11) were initially spontaneously active but after 32 min only 27% (3/11) of neurons were still active]. For control neurons, 80% (8/10) initially displayed tonic firing, with 60% (6/10) still active after 32 min of recording. We examined at what time the neurons stopped spontaneously firing and found that on average control (vehicle) neurons that ceased firing stopped firing at 26 ± 3.7 min, which was similar to the time for neurons that received α-syn monomers (28 ± 3.64 min). The neurons that received α-syn aggregates stopped firing significantly earlier (15.1 ± 2.33 min) than either control (vehicle) neurons (*p* = 0.0274) or neurons that received α-syn monomers (*p* = 0.0392).

### Further analysis of the effects of α-syn aggregates on DNs using the DIV curve

**Figure 5.**
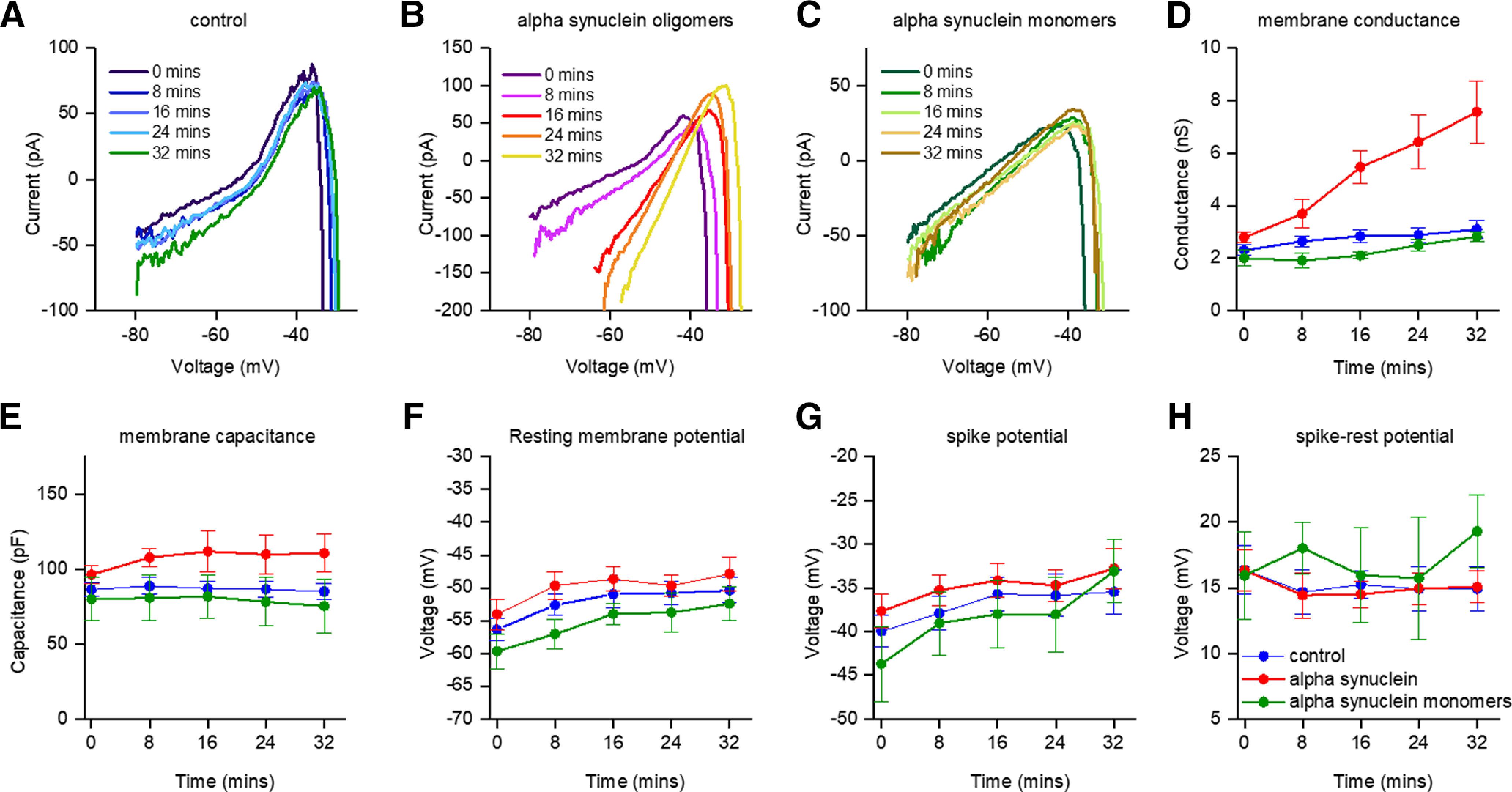
Intracellular diffusion of α-syn aggregates results in a progressive conductance increase in the DIV curve. Example DIV curves from neurons that had control (vehicle, ***A***), α-syn aggregates (***B***), or monomeric α-syn (***C***) introduced. Each graph shows the DIV curve at 8-min time points throughout recordings (32 min). There is a marked increase in the gradient of the DIV (conductance) when oligomeric α-syn was introduced. ***D–H***, Extracted neuronal parameters averaged across recordings for the time periods during recordings (*n* =1 0 for control, *n *=* *11 α-syn aggregates and *n *=* *6 α-syn, monomers). Capacitance (***D***) showed no significant change over time for all the experimental conditions. Although both control (vehicle) and monomeric α-syn neurons showed a small increase in conductance (***E***) over time (∼35% and ∼40% after 32 min, respectively), the average conductance for the cells receiving α-syn aggregates was almost threefold its initial value by 32 min (∼170% increase; *p* = 0.009). At 32 min, there was no significant (*p* > 0.9999) difference between the conductance of vehicle and α-syn monomer treated neurons. In contrast, the conductance of α-syn aggregate treated neurons was significantly (*p* = 0.0003) larger than vehicle treated neurons. Although both the resting potential (***F***) and spike threshold (***G***) slightly increased with time, the potential difference from resting potential to spike threshold remained constant with time. The decrease in excitability of the cells receiving α-syn aggregates was therefore mediated by a conductance shunt rather than an increase in the relative threshold for spike initiation.

In control neurons (vehicle) the DIV curve did not markedly change throughout the duration of recordings (Fig. 5*A*). In contrast α-syn aggregates induced significant changes to the DIV, in particular increasing the slope (Fig. 5*B*). These effects were not observed with α-syn monomers (Fig. 5*C*). For all experimental conditions, there were no significant changes in the cellular capacitance (*p* = 0.18; Fig. 5*E*). Thus, the introduction of α-syn aggregates had no effect on the electrotonic properties of the neurons, by, for example, electrically isolating compartments. Aggregated α-syn markedly increased the membrane conductance (272% of the conductance at 0 min by 32 min, *p* = 0.001; Fig. 5*D*). In control neurons (vehicle or α-syn monomers), there was a small increase in conductance (vehicle conductance was 135% of the conductance at 0 min by 32 min *p* = 0.009; α-syn monomer conductance was 141.2% of the conductance at 0 min by 32 min, ns) over the duration of recordings. At 32 min there was no significant (*p* > 0.9999) difference between the conductance of vehicle and α-syn monomer treated neurons. In contrast, the conductance of α-syn aggregate treated neurons was significantly (*p* = 0.0003) larger than vehicle treated neurons. There were no significant differences in the other extracted parameters: resting membrane potential, spike potential or spike-resting potential between the three experimental conditions (Fig. 5*F–H*). Thus, the reduction in excitability induced by α-syn aggregates is only because of a change in conductance rather than changes in, for example, the potential difference between rest and threshold.

### The decrease in conductance and firing rate caused by α-syn is in part because of the opening of KATP channels

**Figure 6.**
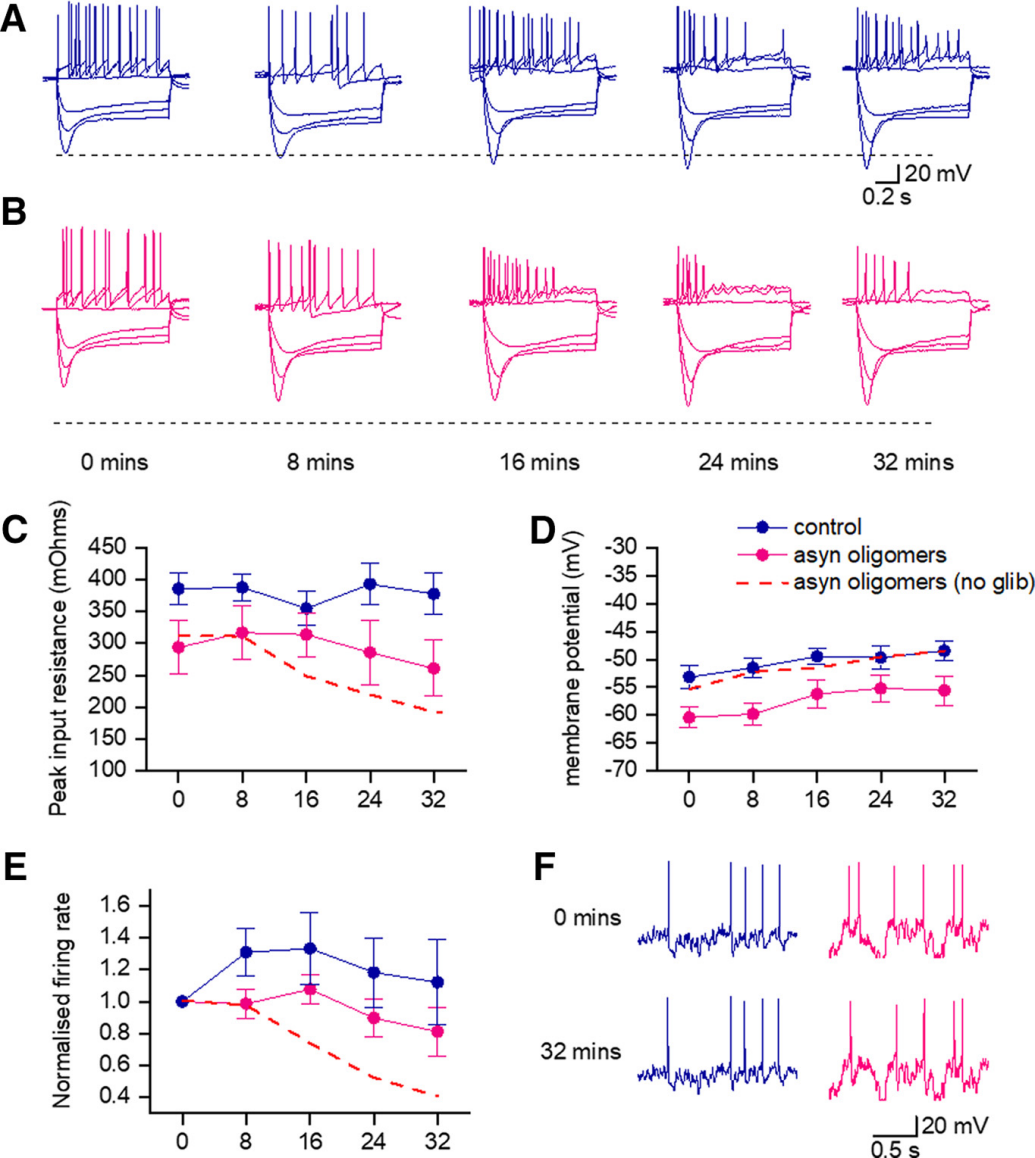
The effects of α-syn aggregates on electrophysiological properties are reversed by glibenclamide. ***A***, SIV for an example control neuron (injected with vehicle) in the presence of the KATP channel inhibitor glibenclamide (1 μm). Current steps (starting at –300 pA and rising by 100 pA) were injected until a regular firing pattern was induced. SIV traces are displayed at time points between whole-cell breakthrough (0 min) and the end of recording (32 min). The neuron remained stable for the duration of the recording with little change in the SIV. ***B***, As in ***A***, but with α-syn aggregates (500 nm, final concentration) added to the internal recording solution. The decrease in input resistance and firing rate that was observed with α-syn aggregates in control conditions was markedly reduced by the presence of glibenclamide**. *C***, Mean resistance measurements over time for control (vehicle) versus α-syn aggregates in the presence of glibenclamide. The fall in resistance was reduced compared with that for α-syn aggregate-injected neurons recorded in normal aCSF. ***D***, Mean resting membrane potential measurements plotted against time for vehicle versus α-syn aggregates in the presence of glibenclamide. ***E***, Mean firing rate (measured from naturalistic current injection) plotted against time for vehicle versus α-syn aggregates in the presence of glibenclamide. The fall in firing rate was reduced compared with that for α-syn aggregate-injected neurons in normal aCSF. The red dotted line represents the effects observed with the introduction of α-syn aggregates in the absence of glibenclamide, these data are repeated from Figure 4 and are intended to provide a visual reference to demonstrate the partial recovery.

Previous studies have shown that either blocking KATP or genetically deleting them has protective effects on DNs in PD rodent models (Liss et al., 2005; Zhang et al., 2018). There has also been a recent preprint that shows that the effects of α-syn aggregates on spontaneous firing can be prevented by blocking KATP channels (Thakur et al., 2019). To investigate whether the increase in membrane conductance that we have observed could be prevented by inhibiting KATP channel opening, we used the classic KATP blocker glibenclamide (Light and French, 1994; Jiang and Haddad, 1997). We first investigated whether the presence of glibenclamide throughout recordings had any effect on the electrophysiological properties of SNpc DNs (Fig. 6*A*). There was no significant difference in any of the measured parameters (from SIVs, membrane potential *p* = 0.3791, resistance *p* = 0.2294, firing rate *p* = 0.6548) at 0 min between neurons from slices which had been preincubated with glibenclamide (1 μm, *n *=* *6) compared with neurons in control slices (both with vehicle in the patch pipette). These neuronal parameters did not significantly change over the duration of the recordings with glibenclamide present: resistance (after 32 min 99 ± 7.5% of the resistance at time 0 min, *p* = 0.08,438, *n *=* *6) or firing rate (after 32 min 112.1 ± 26.6% of the firing rate at time 0 min, *p* = 0.5625; Fig. 6*A*). Thus, glibenclamide had no significant effect on neuronal properties measured from SIVs that could occlude the actions of α-syn aggregates.

We then made whole-cell recordings with α-syn aggregates in the patch pipette in slices preincubated in glibenclamide (1 μm; Fig. 6*B*). The fall in resistance and firing rate that was observed in the presence of α-syn aggregates did not occur when the slices were incubated with glibenclamide. There was no significant reduction in resistance or firing rate over the duration of recording when slices were incubated with glibenclamide suggesting that the action of α-syn aggregates could least in part be mediated by KATP channels. The resistance at 32 min was 90 ± 8.62% of the resistance at time 0 min (compare 63% without glibenclamide), which was not statistically significant (*p* = 0.25, *n *=* *6) from the resistance at time 0. The firing rate was 80.14 ± 12.89% of the rate at time 0 min (vs 42% without glibenclamide) which was not statistically significant from the firing rate at time 0 min (*p* = 0.353, *n *=* *6). Both sets of recorded neurons (vehicle and α-syn aggregates) weakly depolarized over the 32 min of recording to a similar degree to that of non-glibenclamide-treated slices (vehicle, ΔVm 4.66 ± 2.6 mV and α-syn aggregates, ΔVm 5.5 ± 2.05 mV; Fig. 6*E*).

**Figure 7.**
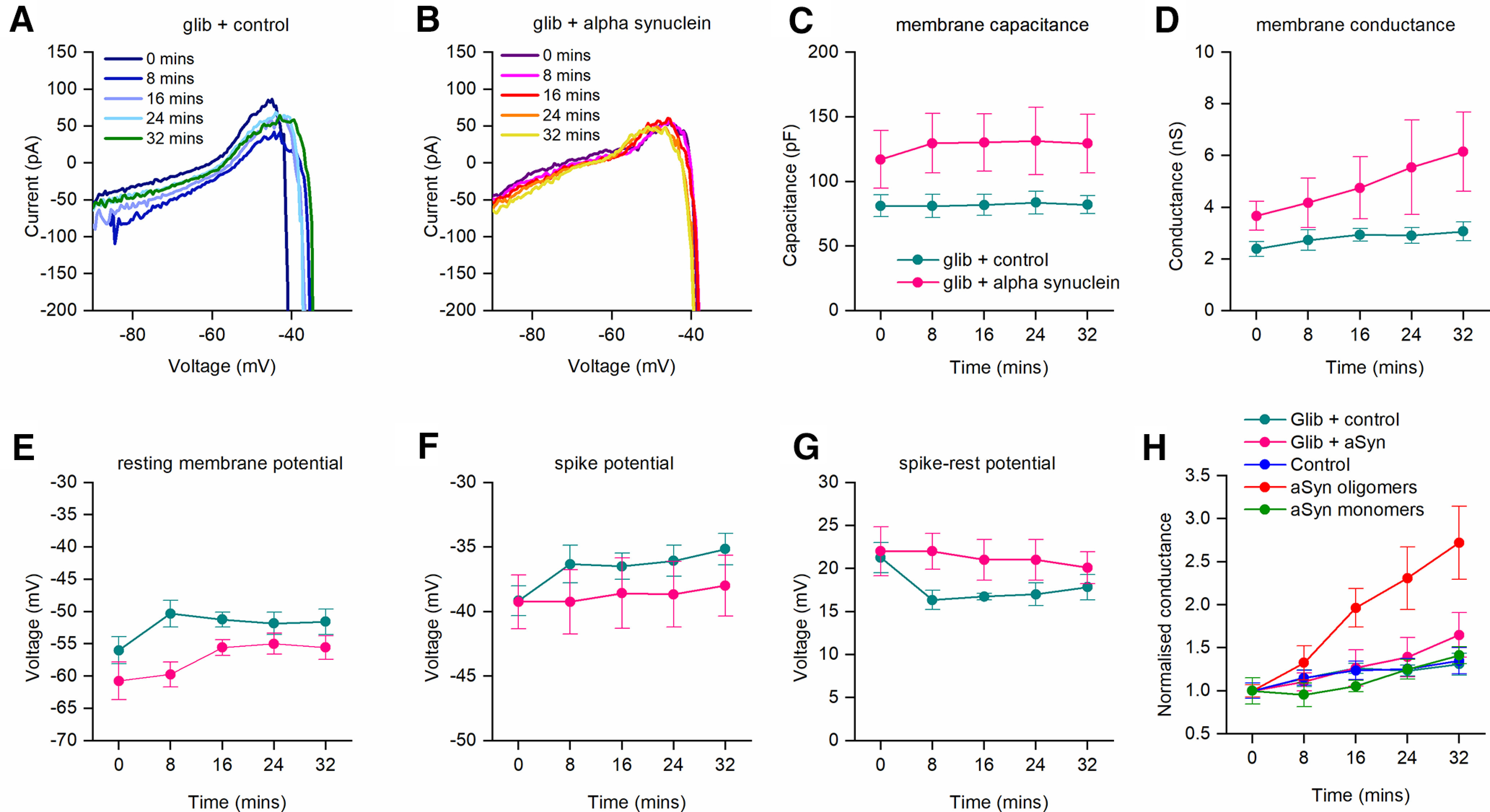
The KATP channel inhibitor glibenclamide blunts the effects of α-syn aggregates on the DIV. Slices were incubated in glibenclamide (1 μm) to block KATP channels. ***A***, Example DIV curves for a neuron that had vehicle introduced. ***B***, Example DIV curves for a neuron that had α-syn aggregates introduced. For ***A***, ***B***, each graph shows the DIV curves at 8-min time points throughout recordings (32 min). There are no marked changes in the DIV across time for the two experimental conditions. ***C–H***, Extracted neuronal parameters averaged across recordings for the time periods during recordings (*n *=* *6 for vehicle, *n *=* *6 α-syn aggregates). Capacitance (***C***) showed no significant change over time for all the experimental conditions. ***D***, There was a small increase in whole-cell conductance for neurons with α-syn aggregates, but the percentage change was closer to vehicle than to α-syn aggregates without glibenclamide. Resting potential (***F***) and spike threshold (***G***) also slightly increased with time, the potential difference from rest potential to spike threshold remained constant with time. ***H***, Graph plotting the normalized conductance (normalized to the conductance at time 0 min) for all experimental conditions. Using a Kruskal–Wallis ANOVA, we confirmed that both time and experimental condition had a significant effect on conductance (*p* < 0.0001 and *p* < 0.0001, respectively). At 32 min, the neurons with α-syn aggregates injected have a significantly greater conductance than any of the other experimental conditions (vs control *p* < 0.0001, vs monomer *p* = 0.0003, vs glib + control *p* < 0.0001, vs glib + α-syn *p* = 0.0045; Dunn’s *post hoc* analysis). No other experimental conditions were significantly different from each other.

We further investigated the effects of glibenclamide on the effects of α-syn aggregates, using the DIV. The DIV curves in glibenclamide (vehicle) and in glibenclamide (α-syn aggregates) did not markedly change throughout the duration of the recordings (Fig. 7*A*,*B*). As observed previously (without glibenclamide) there was also no change in capacitance over the duration of the recordings (vehicle *p* = 0.0625, α-syn aggregates *p* = 0.0625; Fig. 7*C*). In the presence of glibenclamide, there was no significant increase (*p* = 0.0625) in whole-cell conductance for vehicle receiving neurons over the duration of the recordings or only a small increase for α-syn aggregate receiving neurons (*p* = 0.0313). After 32 min, there was no significant (*p* = 0.3095) difference between the whole-cell conductance of neurons receiving vehicle and those receiving α-syn aggregates (Fig. 7*D*,*H*). For all the other extracted parameters there was no difference between vehicle and α-syn aggregate receiving neurons for the duration of recordings (Fig. 7*E–G*). Thus, as observed with SIV analysis, the KATP inhibitor glibenclamide significantly reduced the effects of α-syn aggregates.

## Discussion

In this study, we have used whole-cell patch-clamp recording to introduce a known concentration and form of aggregated α-syn into single SNpc DNs in acutely isolated mouse slices. Although it is possible, using emerging technologies, to isolate only the oligomeric fractions (Kumar et al., 2020), we have not utilized such methods. Thus, we cannot exclude the possibility that some small fibrils are present in our α-syn oligomer samples. While the majority of the species in our α-syn samples appeared to have an oligomeric structure (Fig. 3) other forms of small aggregates maybe present (for example, small ∼50-nm fibrils; Polinski et al. 2018). We have therefore used the term α-syn aggregates rather than α-syn oligomers. As outlined in previous studies (Kaufmann et al., 2016; Hill et al., 2019) delivery of species via the patch pipette has a number of advantages, the main one being that it is possible to measure early changes in the properties of a single neuron in a network that is free from pathology. This method also bypasses any slow uptake steps and each neuron acts as its own control, as at early time points only low concentrations of the species will have diffused into the neuron. Here, we have introduced 500 nm α-syn aggregates, which is a concentration in line with previous studies (see Kaufmann et al., 2016). However, as noted in the methods, the actual concentration of aggregates will be considerably lower. It would be interesting in future experiments to investigate whether there is a concentration dependent effect of α-syn aggregates, with, for example, lower concentrations having a slower onset (see Hill et al., 2019).

We used both SIV (with step current injections) and the DIVs (with naturalistic current injection) to extract neuronal parameters. Both approaches measured a marked increase in whole-cell conductance (fall in neuronal resistance), which occurred between 8 and 16 min after whole-cell break through. There were no significant changes in membrane potential, cell capacitance or spike threshold between neurons receiving α-syn aggregates, α-syn monomers, or vehicle. The increase in conductance significantly reduced the firing rate, both induced (by current injection) and spontaneous, and abolished rebound firing following hyperpolarizing current steps. The increase in conductance increased the linearity of the DIV curve effectively shunting the depolarization-activated conductance increase just below threshold that was present in some neurons at 0 min (whole-cell breakthrough).

Obtaining similar results using two independent methods for the extraction of neuronal parameters strengthens the robustness of the observation that α-syn aggregates increase whole-cell conductance. There were minor differences in the precise values of the neuronal parameters extracted using DIV compared with those extracted with SIV, which is to be expected given the different conductance states of the cell in the two protocols. For the DIV, the voltage varies across a wide range during naturalistic stimulation (mimicking *in vivo* synaptic activity) activating and inactivating a variety of conductances. In contrast, the SIV extracts parameters over a much smaller range of voltages from essentially quiescent cells. For example, if the DIV and SIV curves are compared, the DIV conductances are initially lower (at 0 min) for all of the experimental conditions (vehicle, aggregates and monomers) and then increase. This could be interpreted as one or more of the conductances being slightly more inactivated during the DIV protocol. It could then be that this inactivation is relieved as the neuronal resting potential slowly depolarizes during the recording. Alternatively, it could be that another conductance activates as the neuron depolarizes, but in a range that is not accessed by the hyperpolarizing SIV current steps. In either case, a different range of voltages is probed between DIV and SIV and so differences in the extracted parameters will occur. The fact that the same significant conductance increase (resistance decrease) is clearly seen in these two very different protocols attests to the robustness of the effect of α-syn on these neurons.

### The increase in whole-cell conductance is reduced by the KATP channel blocker glibenclamide

The effects of the KATP inhibitor glibenclamide (Light and French, 1994) suggest the involvement of KATP channels (but see caveats below). KATP channels are inwardly rectifying K^+^-selective ion channels that are inhibited by intracellular ATP. KATP channels provide a link between the energy state of cells and their electrical activity acting as a metabolically controlled “brake on excitation.” A decrease in sub-membrane ATP levels and an accompanying rise in ADP concentration (during activity) triggers KATP channel opening dropping neuronal resistance and hyperpolarizing the membrane potential (Stanford and Lacey, 1995; Seino, 1999; Haller et al., 2001). It is well established that SNpc DNs express KATP channels (Liss et al., 2005; Schiemann et al., 2012). The lack of effect of the KATP inhibitor glibenclamide under control conditions suggests that few KATP channels are open at rest. This is perhaps not surprising as there was 2 mm ATP in the intracellular patch solution, although some KATP channels have a low affinity for ATP and would still be open with this level of ATP (see Allen and Brown, 2004). The effects of α-syn aggregates on conductance and its inhibition by glibenclamide are consistent with the opening of KATP channels (but see below). These effects occurred in the presence of 2 mm intracellular ATP suggesting a direct effect of α-syn aggregates on the KATP channel, rather than via a reduction in intracellular ATP concentration (by, for example, mitochondrial dysfunction). KATP channel openers like diazoxide have previously been shown to act by open KATP channels in the presence of ATP (Schwanstecher et al., 1998) so it is possible that α-syn aggregates could have a similar action.

The effects on cell conductance and its prevention with glibenclamide are consistent with the α-syn aggregates increasing KATP channel open probability. However, there was little or no associated membrane potential hyperpolarization (measured with SIV and with DIV), which has been observed in most reports of KATP channel activation (see Stanford and Lacey, 1995; Allen and Brown, 2004). The equilibrium potential for K^+^ is ∼−95 mV, so opening of KATP channels would be expected to produce a large hyperpolarization of the membrane potential (as the resting potential at time 0 was ∼−55 mV). However, the membrane potential hyperpolarization could possibly be counteracted by the opposing activation of hyperpolarization-activated, cyclic nucleotide-gated HCN channels I(h) which have a reversal potential between −40 to −30 mV (Mayer and Westbrook, 1983). It may be that the opening of a relatively small number of KATP channels changes the input resistance/conductance of neurons but is insufficient to change the membrane potential against the responsive reaction of I(h). In contrast opening of many KATP channels will overcome the effects of I(h) leading to robust membrane hyperpolarization, which as reported for SNpc DNs by lowering intracellular ATP concentration or using KATP channel opening drugs (Stanford and Lacey, 1995).

The inhibitory effects of glibenclamide on α-syn responses are consistent with the opening of KATP channels but additional evidence is required to provide definitive evidence for a role for KATP channels in the effects of α-syn aggregates. One possible approach is to occlude the effects of α-syn aggregates using a pharmacological opener of KATP channels (such as diazoxide). However, these experiments maybe be difficult to interpret. The KATP channel opener will markedly reduce resistance and hyperpolarize the recorded neuron. This may occlude the effects of α-syn aggregates simply by shunting the neuron and moving it closer to E_K_ rather than acting through the same channel. Other possible approaches include the use of transgenic animals, where the KATP gene has been deleted, or using cell lines expressing specific KATP channels. Alternate possibilities for the lack of membrane hyperpolarization include the opening of other channels that depolarize the neuron. For example, it has been reported that α-syn itself can form non-selective cation channels (Mironov, 2015).

It took on average between 8 and 16 min of dialysis for the effects of α-syn aggregates on whole-cell conductance to become significant. It may be that the concentration within the neuron has to reach a sufficient level to open membrane channels. For comparison introduction of aggregated tau (444 nm) into pyramidal neurons started to have effects on the action potential within the first 10 min (Hill et al., 2019). In this study, we chose to focus on the effects of introducing α-syn aggregates into substantia nigra DNs. Given that the neighboring population of VTA DNs have differences in their KATP channel expression (Liss et al., 2005), it might be expected that α-syn aggregates may have a different effect in these cells (which would additional evidence for a role of KATP channels). This may contribute to the varying vulnerability of these two dopaminergic nuclei in diseases like PD.

The accumulation of α-syn aggregates in SN DNs during PD progression could potentially result in the prolonged activation of membrane channels (such as KATP) which will chronically reduce electrical activity, the amount of dopamine released (Patel et al., 2011) and will be detrimental to neuron function. If KATP channels are involved then there will be a loss of the metabolic feedback mechanism. Consistent with this KATP involvement, there is retrospective epidemiological evidence of a reduced risk for PD in type 2 diabetic patients that were treated with KATP inhibitors (Schernhammer et al., 2011; Wahlqvist et al., 2012; Cereda et al., 2013; Lu et al., 2014; Brauer et al., 2015).

In this study, we have combined electrophysiological recording with detailed and thorough analysis to characterize the effects of introducing aggregated α-syn directly into single mouse DNs in the substantia nigra. Aggregated α-syn caused a significant increase in conductance and decrease in firing rate without altering the resting membrane potential, capacitance or spike threshold. Changes to conductance and firing rate occurred 8–16 min after whole-cell breakthrough and were specific to aggregates (they were not observed when monomeric α-syn was introduced). The effects could be prevented by preincubating the slices in KATP inhibitor glibenclamide, despite the high concentration of ATP present in the patch electrode. This suggests that aggregated α-syn may increase the opening probability of KATP channels, resulting in an increase in conductance, a reduction in neuronal excitability and likely also a decrease in dopamine release and overall cell function.

## Synthesis

Reviewing Editor: Mikko Airavaara, University of Helsinki Decisions are customarily a result of the Reviewing Editor and the peer reviewers coming together and discussing their recommendations until a consensus is reached. When revisions are invited, a fact-based synthesis statement explaining their decision and outlining what is needed to prepare a revision will be listed below. The following reviewer(s) agreed to reveal their identity: Andrii Domanskyi.

The reviewers found merit in the study and were worried about the conclusion whether they are valid. Based on my reading of the paper, I do not think that additional experimental data are necessary needed for a successful revision, but it is important to fully address the critical comments of reviewer #1 and #2 regarding experimental asyn molecules used and statistical analysis. I enclose below the individual comments of the reviewers that should be addressed as I have indicated below. In addition to reviewers comments the revised manuscript should be strictly in the format of eNeuro including the sections related to statistics and stastitical table, RRIDs, eNeuro citing, see https://www.eneuro.org/content/preparing-manuscript#organization . Also n number should be clearly written in each figure legend.

I think addressing these will made the study substantially better.

Reviewer #1

The manuscript “Alpha-synuclein oligomers significantly increase the resting conductance of substantia nigra dopamine neurons via KATP channel activation” covers important and highly debated research topic i.e., the mechanism by which alpha-synuclein aggregation contributes to dysfunction and deterioration of dopaminergic system seen in Parkinson’s Disease.

Utilizing advanced experimental setup, the authors demonstrate interesting effects on electrophysiological properties of dopamine neurons, which certainly would be of interest to the field of Parkinson’s Disease research. However, there are two significant flaws which need to be addressed before the manuscript could be considered fit for publication: the information on alpha-synuclein molecules utilized in the study is mostly lacking, and the support for proposed KATP channel-dependent mechanism is insufficient for bold conclusions of the authors.

The authors need to address the following issues before the manuscript could be reconsidered for publication:

Major:

1) The authors’ description of injected alpha-synuclein molecules needs to be expanded in several aspects. The authors claim that they inject alpha-synuclein oligomers. The authors insuficciently describe procedure how oligomers were obtained from long alpha-synuclein fibrils which were the initial material. What were the sonication parameters? Has the sample been processed additionally to ensure that only oligomers are present, for example like in Kumar et al. 2020?

Please provide additional support that only oligomers and not other forms of aggregates (i.e. fibrils) were injected into neurons.

Sonicated long alpha-synuclein fibrils would usually shatter to shorter fibrils and it remains unclear if and how much of alpha-synuclein would become oligomers for example trough dissociation from short fibrils. Most studies demonstrate that after sonication long aSyn PFFs became short (∼50nm) fibrils (See Polinski et al 2018 and others). Provided EM image don’t show small fibrils, however, this can be hard to see depending on the staining procedure.

Lastly, please detail how the concentration of injected alpha-synuclein was calculated - the authors use molar concentration but how can this be obtained without precisely knowing molar mass of alpha-synuclein molecules? Is this molar concentration of monomer units?

If there is a possibility that not only oligomers were utilized in “oligomer” groups, authors should use the broader term (e.g. alpha-synuclein aggregates) and discuss this limitation accordingly.

Kumar, S. T., Donzelli, S., Chiki, A., Syed, M. M. K., & Lashuel, H. A. (2020). A simple, versatile and robust centrifugation-based filtration protocol for the isolation and quantification of α-synuclein monomers, oligomers and fibrils: Towards improving experimental reproducibility in α-synuclein research. Journal of Neurochemistry. https://doi.org/10.1111/jnc.14955

Editor: should be addressed:

2) The authors claim that observed effects were mediated through KATP channel. However, the evidence for this is not convincing. As authors discussed, there is no hyperpolarization of the membrane, which would be expected if alpha-synuclein “oligomers” opened the channel.

The authors claim that “Glibenclamide significantly reduced both the fall in input resistance (Fig 6C) and firing rate (Fig 6D) induced by the alpha-syn oligomers measured with SIV curves.” Where is the statistic supporting this claim? What is the dotted curve in figures 6 C,D,E ? It does not have any measure of variation - is this data from the glibenclamide experiment, or is it the same curve as in fig 4 reused? Lack of significant difference between control and alpha-syn oligomers+ glibenclamide without a significant difference to no-glibenclamide alpha-synuclein oligomers data is insufficient to support strong statements by the authors.

Either improving presentation of glibenclamide data and providing convincing statistical evidence or additional experiments (e.g. with KATP KO, some biochemical evidence) should be provided. The inclusion of positive control - pharmacological KATP channel opener would benefit the study. Moreover, cotreatment with pharmacological KATP opener together with alpha-synuclein “oligomers” could provide additional support for KATP channel involvement.

Alternatively, authors should considerably tone down claims of the underlying mechanism, including rewriting the manuscript title.

Editor: should be addressed:

Minor:

3) please mark significant differences discussed in the text on the graphs where possible.

Editor: should be addressed.

4) In the introduction, the authors write that oligomers are the most toxic species of alpha-synuclein. “However it is the small soluble oligomers that appear to be the most toxic of the forms of alpha- syn within this aggregation cascade (Winner et al, 2011)”. This is hovever not evident from literature and alternatives have been suggested for example see data from Mahul-Mellier et al. 2020.

Mahul-Mellier, A.-L., Burtscher, J., Maharjan, N., Weerens, L., Croisier, M., Kuttler, F., Leleu, M., Knott, G., & Lashuel, H. A. (2020). The process of Lewy body formation, rather than simply alpha-synuclein fibrillization, is the major driver of neurodegeneration in synucleinopathies. Proc. Natl. Acad. Sci. USA.

Editor: should be addressed.

5) In methods - statistics: authors write that single data points are individual experiments. Could the authors provide information in the manuscript how many different biological repeats i.e. animals, were used? Were data points from the same treatment group always from different animals or not? When different treatment groups were measured in slices from same animal was this considered in the statistic calculations by use of specific matching like in randomized block ANOVA design (see Lew 2007)?

Lew, M. (2007). Good statistical practice in pharmacology Problem 2. British Journal of Pharmacology, 152(3), 295-298. https://doi.org/10.1038/sj.bjp.0707370

Editor: should be addressed.

6) On page 12 the authors write: “firing rate at 32 mins was 88.5 {plus minus} 7.4 % of % of the firing rate at time 0 mins, n = 10, Fig 4F, G)” but it looks rather like ∼120% similar to monomer group.

Editor: should be addressed.

7) On page 13 the authors write: “In contrast, all the control neurons that initially displayed rebound firing (60 %) still showed it after 32 minutes of recording. Oligomeric alpha-syn also reduced the occurrence of tonic firing (64 % of neurons were initially spontaneously active but after 32 minutes only 27 % of neurons were still active). For control neurons, 80 % initially displayed tonic firing, with 60% still active after 32 minutes of recording.” This data should be put into table or otherwise visualized with a specific number of neurons provided. The differences could also be statistically tested.

Editor: should be addressed.

8) On page 14: Fig 5D and Fig 5E are mixed

Editor: should be addressed.

9) On page 15: “firing rate (Fig 6D)” refer to fig 6E. Also entire sentence is not supported by statistics, see Major comment 2.

Editor: should be addressed.

10) On page 15: The authors write: “There was a small but significant (P = 0.0313) increase in whole cell conductance for vehicle receiving neurons over the duration of the recordings but not for the alpha-syn oligomer receiving neurons (P = 0.0625).” that is true, but alpha-syn “oligomer” group had tremendous variation suggesting that experient had some technical problems which do not allow for clear conclusions.

Editor: should be addressed.

Reviewer 2

In their manuscript, the authors have addressed the effects of oligomeric alpha-synuclein on electrophysiological properties of nigral dopaminergic neurons. They convincingly demonstrate that alpha-synuclein oligomers, but not monomers, are causing a significant increase in conductance and decrease in firing rate; this effect is apparently due to increasing the opening probability of KATP channels. These very interesting and novel results are advancing our understanding of alpha-synuclein functions and biology of dopaminergic neurons, which are crucial also for the development of new disease-modifying therapies for Parkinson’s disease.

The manuscript is very well written; however, several following points need to be clarified.

It is known that oligomers’ properties strongly depend on the preparation protocol; however, it is not sufficiently described in the Methods section. Specifically, which exactly monomers, oligomers and fibrils have been used? The authors state that “...Recombinant human alpha-synuclein aggregates were purchased from Abcam (ab218819) along with the corresponding monomers (ab218817).” But ab218817 product is “Recombinant Human Alpha-synuclein protein aggregate (Type 2)”. Are these products represent alpha-synuclein fibrils (as seen in Fig. 3B), which then been sonicated by the authors to obtain oligomers and monomers (Fig. 3C and 3D)? If not, how fibrillizaton was achieved? Which sonication parameters have been used? Despite the authors’ mentioning this in the text, I could not find detailed information in the Methods section. This needs to be clarified.

Editor: should be addressed.

The images on Fig. 1F do not clearly show co-localization of AF594 with TH immunostaining. In fact, the AF594/TH signals appear to me as not co-localizing. To clarify the issue, the cells can be marked by, for example, arrowheads in TH channel.

Editor: should be addressed.

Though it has been addressed in the Discussion section, from the data presented in Fig. 2 it is not immediately clear, if the two methods (SIV and DIV) used by the authors to characterize the capacitance and conductance provide comparable results. Can the authors explicitly show the estimated values for the capacitance and conductance derived from the data on Fig. 3A-C and Fig. 3D-F?

Editor: should be addressed.

The concentration of alpha-synuclein oligomers used in the study (500 nM) is rather high. Have the authors tried different concentrations? Demonstrating concentration-dependent effect of alpha-synuclein oligomers on measured electrophysiological properties would, in my view, significantly strengthen the manuscript conclusions. It is also rather unfortunate that the authors focused solely on nigral dopaminergic neurons. It would have been extremely interesting to compare the effects of alpha-synuclein oligomers on nigral vs VTA dopaminergic neurons, especially as activation of KATP channels in these neuronal populations is known to be different (Liss et al, 2005). VTA neurons are relatively spared in Parkinson’s disease patients, so it would have been exceptionally interesting to study if they respond to alpha-synuclein oligomers differently from nigral population. The nigral and VTA dopaminergic neurons are anatomically very close and, most probably, present on the same brain slice (depending on the preparation); the authors would have all technical capacity to analyze and compare these two populations.

Editor: should be addressed, and also how concentrations was calculated should written out . If new experiments are not possible in the current situation it is enough to address these in the discussion.

Panels on Fig. 5D and 5E appear to be mixed - in the text (page 14), Fig. 5D is referred to as showing cellular capacitance and Fig. 5E - conductance, whereas in the figure itself they are other way round.

Editor: should be addressed.

Legend to Fig. 6 (explaining color codes; shown on panel D) is rather misleading. According to the manuscript text and figure caption, the samples have been treated with Glibenclamide and, therefore, “control” should be “Glib + control” and “asyn oligomers” should be “Glib + asyn oligomers”.

Editor: should be addressed.

Comparing Fig. 7B with Fig. 5E (showing membrane capacitance), if seems that glibenclamide treatment in combination with alpha-synuclein oligomers, in comparison with glibenclamide + control, has increased membrane capacitance throughout the whole experimental timeline. Similarly, on Fig. 7D the membrane conductance after glib + alpha-synuclein treatment appears to be increased (as compared to glib + control). Surprisingly, these changes are already present at time point 0, so both curves are shifted up compared to control. Does this reflect differences in baseline parameters due to, for example, different slice preparations? If so, why the baseline parameters in samples shown on Fig. 5D, E are more consistent (i.e. the curves start from approximately the same value at t=0, as would be expected)?

Editor: should be addressed.

## Author Response

Reply to Editor and reviewer comments

We thank the reviewers and editor for their comments which will improve the final version of the manuscript. We have amended the text, carried out reanalysis of the data, provided additional statistics, added references and have changed some of the figures as requested.

Editor comment 1. The Visual Abstract for Author (Required) is missing and needed.

We apologise for not submitting this in the first round, we have included this in the uploaded files for the revision.

Editor comment 2. “The reviewers found merit in the study and were worried about the conclusion whether they are valid. Based on my reading of the paper, I do not think that additional experimental data are necessary needed for a successful revision, but it is important to fully address the critical comments of reviewer #1 and #2 regarding experimental asyn molecules used and statistical analysis. I enclose below the individual comments of the reviewers that should be addressed as I have indicated below. In addition to reviewers comments the revised manuscript should be strictly in the format of eNeuro including the sections related to statistics and stastitical table, RRIDs, eNeuro citing, see https://www.eneuro.org/content/preparing-manuscript#organization . Also n number should be clearly written in each figure legends"

We have taken care to address each reviewer comment point-by-point as outlined below.

Response to comments of Reviewer 1

Point 1.1a “The authors’ description of injected alpha-synuclein molecules needs to be expanded in several aspects. The authors claim that they inject alpha-synuclein oligomers. The authors insufficiently describe procedure how oligomers were obtained from long alpha-synuclein fibrils which were the initial material. What were the sonication parameters? Has the sample been processed additionally to ensure that only oligomers are present, for example like in Kumar et al. 2020?"

We agree that there could be more detail regarding the alpha synuclein species used in our experiments. We have added this additional information to the methods/results sections:

Alpha synuclein aggregates were commercially obtained from Abcam (ab218819). The aggregates were initially characterised (with EM) and confirmed to be pre-formed fibrils (PFFs). We used the methods outlined in Polinski et al (2018) to produce oligomers. Polanski (2018) showed that sonication of PFFs produces a mixture of short (∼50nm) fibrils and oligomers. Our sonication protocol was 15 minutes sonication (50-60 Hz) using a Grant Ultrasonic XUBA1 bath. We then performed EM to examine the structure of the aggregates. We did this upto 3 hours post sonication, after storage on ice, so we could check the structure of the aggregates over the time taken for experiments. The aggregates appeared to have an oligomeric structure (comparable to tau oligomers).

Point 1.1b “Please provide additional support that only oligomers and no other forms of aggregates (i.e. fibrils) were injected into neurons.” and “Sonicated long alpha-synuclein fibrils would usually shatter to shorter fibrils and it remains unclear if and how much of alpha-synuclein would become oligomers for example trough dissociation from short fibrils. Most studies demonstrate that after sonication long aSyn PFFs became short (∼50nm) fibrils (See Polinski et al 2018 and others). Provided EM image do not show small fibrils, however, this can be hard to see depending on the staining procedure.”

Although the majority of the species in our samples appear to be oligomeric (from EM), we cannot exclude the possibility some small fibrils are present. We have clarified this in the revised manuscript by adding a sentence to the results:

“As we cannot exclude the possibility that our samples of alpha synuclein contain some short fibrils, we have used the term alpha synuclein aggregates rather than oligomers”.

Point 1.1c “Lastly, please detail how the concentration of injected alpha-synuclein was calculated - the authors use molar concentration but how can this be obtained without precisely knowing molar mass of alpha-synuclein molecules? Is this molar concentration of monomer units?"

We thank the reviewer for raising this point as it is an important clarification. Our molar concentrations are based on the molar mass of monomeric alpha-synuclein due to samples potentially containing a range of aggregate sizes. This is a common method used in similar studies (see Hill et al, 2019 and Thakur et al, 2019). We have added this to the methods section of the revision.

Point 1.1d If there is a possibility that not only oligomers were utilized in “oligomer” groups, authors should use the broader term (e.g. alpha-synuclein aggregates) and discuss this limitation accordingly.

Answered above, we have added full discussion of this with reference to Kumar’s paper which has been added to the list of references. As outlined above, we have used the term alpha-synuclein aggregates in the revised paper.

Point 1.2a “The authors claim that observed effects were mediated through KATP channel. However, the evidence for this is not convincing. As authors discussed, there is no hyperpolarization of the membrane, which would be expected if alpha-synuclein “oligomers” opened the channel.”

The evidence for the involvement of KATP channels stems from the inhibitory effect of glibenclamide, a well characterised KATP channel inhibitor. However the lack of membrane hyperpolarisation suggests that perhaps other channels are also involved or even a channel other than KATP, which is blocked by glibenclamide, produces the effects. Another possibility (as outlined in the manuscript) is that only a small number of KATP channels open and this does not change membrane potential (perhaps counteracted by IH).

As we have not shown conclusively that KATP channels are involved in the effects of αsyn aggregates we have changed the title of the manuscript, the results and the discussion sections.

Point 1.2b “The authors claim that “Glibenclamide significantly reduced both the fall in input resistance (Fig 6C) and firing rate (Fig 6D) induced by the alpha-syn oligomers measured with SIV curves.” Where is the statistic supporting this claim?"

We examined the relative change over the time 0-32 mins for αSyn vs αSyn in the presence of glibenclamide (using Mann-Whitney):

• a p value of 0.0444 for change in input resistance

• a p value of 0.0254 for change in firing rate

This has been added to the results.

Point 1.2c “What is the dotted curve in figures 6 C,D,E ? It does not have any measure of variation - is this data from the glibenclamide experiment, or is it the same curve as in fig 4 reused? Lack of significant difference between control and alpha-syn oligomers+ glibenclamide without a significant difference to no-glibenclamide alpha-synuclein oligomers data is insufficient to support strong statements by the authors.”

The dotted line in Figures 6 C, D, E represents the effects of alpha synuclein without the presence of glibenclamide. The reviewer correctly ascertains that this is the same curve from figure 4. It is there to provide a visual reference to show the partial recovery with glibenclamide. It was not included with points and errors) as the data has already been displayed in Figure 4. We have made this clear in the legend

Point 1.2d “Either improving presentation of glibenclamide data and providing convincing statistical evidence or additional experiments (e.g. with KATP KO, some biochemical evidence) should be provided. The inclusion of positive control - pharmacological KATP channel opener would benefit the study. Moreover, cotreatment with pharmacological KATP opener together with alpha-synuclein “oligomers” could provide additional support for KATP channel involvement.”

While we appreciate suggestions for additional experiments and can see the merit in testing pharmacological KATP channel openers, given the current situation, additional experiments are not possible. It would also be difficult to interpret the data from such experiments. A KATP channel opener would be expected to reduce the input resistance and hyperpolarise neurons. This may occlude the effects of α-syn aggregates simply by shunting the neuron and moving it closer to EK rather than working through the same channel.

We have added additional discussion on the limitations of our data and discussed possible future experiments.

Point 1.2e “Alternatively, authors should considerably tone down claims of the underlying mechanism, including rewriting the manuscript title.”

In the revised manuscript we have toned down some of the statements on the involvement of KATP channels and have changed the title.

Point 1.3 “please mark significant differences discussed in the text on the graphs where possible.”

We included all statistics in the figure legends to ensure they are clear.

Point 1.4 “In the introduction, the authors write that oligomers are the most toxic species of alpha-synuclein. “However it is the small soluble oligomers that appear to be the most toxic of the forms of alpha- syn within this aggregation cascade (Winner et al, 2011)”. This is hovever not evident from literature and alternatives have been suggested for example see data from Mahul-Mellier et al. 2020.”

We have reviewed our use of references to ensure we are up to date and have added the references suggested.

Point 1.5 “In methods - statistics: authors write that single data points are individual experiments. Could the authors provide information in the manuscript how many different biological repeats i.e. animals, were used? Were data points from the same treatment group always from different animals or not? When different treatment groups were measured in slices from same animal was this considered in the statistic calculations by use of specific matching like in randomized block ANOVA design (see Lew 2007)?"

All of the experiments were carried out using multiple animals. Experiments were interleaved. On each experimental day at least 1 control experiment (vehicle or monomer) was performed along with a mixture of the other experimental conditions. Recordings were made from only one neuron per slice. Data points for each condition were obtained from a minimum of 4 animals.

The exact numbers of animals have been added to the legend for the relevant figures.

Point 1.6 “On page 12 the authors write: “firing rate at 32 mins was 88.5 {plus minus} 7.4 % of % of the firing rate at time 0 mins, n = 10, Fig 4F, G)” but it looks rather like ∼120% similar to monomer group.”

We thank the reviewer for highlighting this, we have reviewed this as it is clearly a typing error that we had overlooked on initial submission. This has been corrected.

Point 1.7 “On page 13 the authors write: “In contrast, all the control neurons that initially displayed rebound firing (60 %) still showed it after 32 minutes of recording. Oligomeric alpha-syn also reduced the occurrence of tonic firing (64 % of neurons were initially spontaneously active but after 32 minutes only 27 % of neurons were still active). For control neurons, 80 % initially displayed tonic firing, with 60% still active after 32 minutes of recording.” This data should be put into table or otherwise visualized with a specific number of neurons provided. The differences could also be statistically tested.”

We do not feel that this data needs to be in a table. However, we will add specific neuron numbers to the results text.

Point 1.8 “On page 14: Fig 5D and Fig 5E are mixed"

This has been corrected in the revised manuscript.

Point 1.9 “On page 15: “firing rate (Fig 6D)” refer to fig 6E. Also entire sentence is not supported by statistics, see Major comment 2.”

This has been rewritten to improve clarity and the statistics added.

Point 1.10 “On page 15: The authors write: “There was a small but significant (P = 0.0313) increase in whole cell conductance for vehicle receiving neurons over the duration of the recordings but not for the alpha-syn oligomer receiving neurons (P = 0.0625).” that is true, but alpha-syn “oligomer” group had tremendous variation suggesting that experient had some technical problems which do not allow for clear conclusions.”

This point refers to the glibenclamide data analysis. There was significant variation in the absolute capacitance and conductance of the cells (due to one large cell in the glib + αsyn data). However, the key point is the change in parameters over the time period 0 to 32mins. Please see our response to Point 2.8.

-

Response to comments of Reviewer 2

Point 2.1 “It is known that oligomers’ properties strongly depend on the preparation protocol; however, it is not sufficiently described in the Methods section. Specifically, which exactly monomers, oligomers and fibrils have been used? The authors state that “...Recombinant human alpha-synuclein aggregates were purchased from Abcam (ab218819) along with the corresponding monomers (ab218817).” But ab218817 product is “Recombinant Human Alpha-synuclein protein aggregate (Type 2)”. Are these products represent alpha-synuclein fibrils (as seen in Fig. 3B), which then been sonicated by the authors to obtain oligomers and monomers (Fig. 3C and 3D)? If not, how fibrillization was achieved? Which sonication parameters have been used? Despite the authors’ mentioning this in the text, I could not find detailed information in the Methods section. This needs to be clarified.”

This has been discussed in response to reviewer 1 earlier (Point 1.1a). We will ensure that the preparation protocol is clear in both the methods and the text of the revision. The product number for the monomers is incorrect (typing error). This has been corrected: Recombinant Human Alpha-synuclein protein monomer (Active) (ab218818).

Point 2.2 “The images on Fig. 1F do not clearly show co-localization of AF594 with TH immunostaining. In fact, the AF594/TH signals appear to me as not co-localizing. To clarify the issue, the cells can be marked by, for example, arrowheads in TH channel.”

In the revised manuscript we have added arrow heads to the figure to ensure that this is clear.

Point 2.3 “Though it has been addressed in the Discussion section, from the data presented in Fig. 2 it is not immediately clear, if the two methods (SIV and DIV) used by the authors to characterize the capacitance and conductance provide comparable results. Can the authors explicitly show the estimated values for the capacitance and conductance derived from the data on Fig. 3A-C and Fig. 3D-F?"

We assume that the referee is referring to figure 4 here rather than 3? In any case, the SIV and DIV protocols were chosen specifically because they are distinct - the cells are in very different conductance states (i.e. just below rest under static current injection or fluctuating/firing, respectively). These distinct protocols are likely to result in slightly different absolute conductances for the same cell; however, that very similar relative effect on the conductance (from the DIV slope) and resistance (from maximum voltage deflection - made clear in updated figure 2) provides a robust test of the generality of the effect of alpha synuclein in different neuronal states. As a comparison the mean conductances from SIV vs DIV (nS) at time zero are vehicle 2.95 vs 2.29; aggregates 3.3 vs 2.78; monomers 2.78 vs 1.99; glib 2.59 vs 2.39; glib + aggregates 3.40 vs 3.67. A discussion of the differences between the SIV and DIV is provided in the revised manuscript. The capacitance (calculated from DIV) did not change over time for any of the experimental conditions (showing the stability of recordings) and so was not considered further

Point 2.4 “The concentration of alpha-synuclein oligomers used in the study (500 nM) is rather high. Have the authors tried different concentrations? Demonstrating concentration-dependent effect of alpha-synuclein oligomers on measured electrophysiological properties would, in my view, significantly strengthen the manuscript conclusions.”

We appreciate the comment that the concentration appears quite high, however it is in line with previous studies (see Kaufmann et al, 2016). Note that the concentration of aggregates used in our experiments is calculated based on the molecular mass of monomeric alpha synuclein (see response to reviewer 1 point 1.1c). Therefore the actual concentration of aggregates will be considerably lower, as the aggregates will have an increased molecular weight compared to the monomers. For example, if the aggregates were tetramers then the concentration used will be 4x smaller. Due to the variation of aggregate size, we decided to report the concentration based on the monomeric molecular weight. This is inline with similar studies (see Hill et al, 2019).

We have added full details of how we calculated the aggregate concentration in the revised manuscript.

Point 2.5 “It is also rather unfortunate that the authors focused solely on nigral dopaminergic neurons. It would have been extremely interesting to compare the effects of alpha-synuclein oligomers on nigral vs VTA dopaminergic neurons, especially as activation of KATP channels in these neuronal populations is known to be different (Liss et al, 2005). VTA neurons are relatively spared in Parkinson’s disease patients, so it would have been exceptionally interesting to study if they respond to alpha-synuclein oligomers differently from nigral population. The nigral and VTA dopaminergic neurons are anatomically very close and, most probably, present on the same brain slice (depending on the preparation); the authors would have all technical capacity to analyze and compare these two populations. Editor: should be addressed, and also how concentrations was calculated should written out . If new experiments are not possible in the current situation it is enough to address these in the discussion.”

We agree that assessing whether there is a concentration dependant effect would be interesting, however we are not in a position to be able to perform additional experiments under the current conditions.

We have added this to the discussion “that changing the concentration of α-syn introduced may change the time course of the effects, as observed in other studies”.

The effects on VTA dopaminergic neurons would form part of an additional study. Again we will add a point to the discussion that different dopaminergic neurons that express different complements of channels may show different sensitivities to α-syn aggregates and this may play a role in their susceptibility.

Point 2.6 “Panels on Fig. 5D and 5E appear to be mixed - in the text (page 14), Fig. 5D is referred to as showing cellular capacitance and Fig. 5E - conductance, whereas in the figure itself they are other way round.”

We have corrected this.

Point 2.7 “Legend to Fig. 6 (explaining color codes; shown on panel D) is rather misleading. According to the manuscript text and figure caption, the samples have been treated with Glibenclamide and, therefore, “control” should be “Glib + control” and “asyn oligomers” should be`Glib + asyn oligomers’.”

We have corrected this.

Point 2.8 “Comparing Fig. 7B with Fig. 5E (showing membrane capacitance), if seems that glibenclamide treatment in combination with alpha-synuclein oligomers, in comparison with glibenclamide + control, has increased membrane capacitance throughout the whole experimental timeline. Similarly, on Fig. 7D the membrane conductance after glib + alpha-synuclein treatment appears to be increased (as compared to glib + control). Surprisingly, these changes are already present at time point 0, so both curves are shifted up compared to control. Does this reflect differences in baseline parameters due to, for example, different slice preparations? If so, why the baseline parameters in samples shown on Fig. 5D, E are more consistent (i.e. the curves start from approximately the same value at t=0, as would be expected)?"

For the current study the key point is the relative change in cellular parameters between the 0 to the 32-minute mark, rather than the absolute value of capacitance or conductance at 0 mins: this is reflected in the normalised changes in 7H. Nevertheless, we thank the reviewers for raising the issue of greater variability (in this and Point 1.10) and we investigated why the mean parameter values at 0 mins were different between α-syn + glibenclamide and the rest of the data. We went back through the computer code and rechecked all the numerical DIV analysis (for all the protocols). Regrettably, an algorithmic step for the removal of electrode-filtering artefacts (required for rapidly fluctuating current - see Appendix of Badel et al 2008) was missed for both the glibenclamide control and glibenclamide α-syn analyses (only). This has now been performed and the revised paper updated. The new conductances for glibenclamide + α-syn and glibenclamide + vehicle are 2.4nS and 3.7nS and the capacitance 117pF and 81pF, respectively. Though now slightly lower, the capacitance for glibenclamide + α-syn still remains somewhat higher than for other protocols. On cell-by-cell examination of the data this was due to one cell being significantly larger than the others (this is reflected in the larger standard deviation of that data, 58pF). The cell was an outlier in absolute value, but not in the key aspect of relative change between 0 and 32mins, so we have chosen to retain the cell in the data analysed.

## References

[B1] Alderson TR, Markley JL (2013) Biophysical characterization of α-synuclein and its controversial structure. Intrinsically Disord Proteins 1:18–39. 10.4161/idp.26255 24634806 PMC3908606

[B2] Allen TG, Brown DA (2004) Modulation of the excitability of cholinergic basal forebrain neurones by KATP channels. J Physiol 554:353–370. 10.1113/jphysiol.2003.055889 14578474 PMC1664773

[B3] Badel L, Lefort S, Brette R, Petersen C, Gerstner W, Richardson M (2008a) Dynamic I-V curves are reliable predictors of naturalistic pyramidal-neuron voltage traces. J Neurophysiol 99:656–666. 10.1152/jn.01107.2007 18057107

[B4] Badel L, Lefort S, Berger T, Petersen C, Gerstner W, Richardson M (2008b) Extracting non-linear integrate-and-fire models from experimental data using dynamic I–V curves. Biol Cybern 99:361–370. 10.1007/s00422-008-0259-4 19011924 PMC2798053

[B5] Bernheimer H, Birkmayer W, Hornykiewicz O, Jellinger K, Seitelberger F (1973) Brain dopamine and the syndromes of Parkinson and Huntington. Clinical, morphological and neurochemical correlations. J Neurol Sci 20:415–455. 10.1016/0022-510x(73)90175-5 4272516

[B6] Bengoa-Vergniory N, Roberts R, Wade-Martins R, Alegre-Abarrategui J (2017) Alpha-synuclein oligomers: a new hope. Acta Neuropathol 134:819–838. 10.1007/s00401-017-1755-1 28803412 PMC5663814

[B7] Bezanson J, Edelman A, Karpinski S, Shah VB (2017) Julia: a fresh approach to numerical computing. SIAM Rev 59:65–98. 10.1137/141000671

[B8] Brauer R, Bhaskaran K, Chaturvedi N, Dexter DT, Smeeth L, Douglas I (2015) Glitazone treatment and incidence of Parkinson's disease among people with diabetes: a retrospective cohort study. PLoS Med 12:e1001854. 10.1371/journal.pmed.1001854 26196151 PMC4511413

[B9] Burré J, Sharma M, Tsetsenis T, Buchman V, Etherton MR, Südhof TC (2010) alpha synuclein promotes SNARE-complex assembly in vivo and in vitro. Science 329:1663–1667. 10.1126/science.1195227 20798282 PMC3235365

[B10] Cereda E, Barichella M, Pedrolli C, Klersy C, Cassani E, Caccialanza R, Pezzoli G (2013) Diabetes and risk of Parkinson's disease. Mov Disord 28:257. 10.1002/mds.25211 23032425

[B11] Damier P, Hirsch EC, Agid Y, Graybiel AM (1999) The substantia nigra of the human brain. II. Patterns of loss of dopamine-containing neurons in Parkinson’s disease. Brain 122:1437–1448. 10.1093/brain/122.8.1437 10430830

[B12] Grace AA, Onn SP (1989) Morphology and electrophysiological properties of immunocytochemically identified rat dopamine neurons recorded in vitro. J Neurosci 9:3463–3481. 2795134 10.1523/JNEUROSCI.09-10-03463.1989 PMC6569889

[B13] Haller M, Mironov SL, Karschin A, Richter DW (2001) Dynamic activation of K(ATP) channels in rhythmically active neurons. J Physiol 537:69–75. 10.1111/j.1469-7793.2001.0069k.x 11711562 PMC2278932

[B14] Harris NC, Constanti A (1995) Mechanism of block by ZD 7288 of the hyperpolarization-activated inward rectifying current in guinea pig substantia nigra neurons in vitro. J Neurophysiol 74:2366–2378. 10.1152/jn.1995.74.6.2366 8747199

[B15] Harrison PM, Badel L, Wall MJ, Richardson MJE (2015) Experimentally verified parameter sets for modelling heterogeneous neocortical pyramidal-cell populations. PLoS Comput Biol 11:e1004165. 10.1371/journal.pcbi.1004165 26291316 PMC4546387

[B16] Hill E, Karikari TK, Moffat G, Richardson MJE, Wall MJ (2019) Introduction of tau oligomers into cortical neurons alters action potential dynamics and disrupts synaptic transmission and plasticity. eNeuro 6:ENEURO.0166-19.2019. 10.1523/ENEURO.0166-19.2019 PMC6794083 31554666

[B17] Iwai A, Masliah E, Yoshimoto M, Ge N, Flanagan L, de Silva HA, Kittel A, Saitoh T (1995) The precursor protein of non-A beta component of Alzheimer's disease amyloid is a presynaptic protein of the central nervous system. Neuron 14:467–475. 10.1016/0896-6273(95)90302-x 7857654

[B18] Jakes R, Spillantini MG, Goedert M (1994) Identification of two distinct synucleins from human brain. FEBS Lett 345:27–32. 10.1016/0014-5793(94)00395-5 8194594

[B19] Kaufmann TJ, Harrison P, Richardson MJE, Pinheiro TJT, Wall MJ (2016) Intracellular injection of soluble alpha synuclein oligomers reduces pyramidal cell input resistance and firing rate. J Physiol 594:2751–2772. 10.1113/JP271968 26915902 PMC4865569

[B20] Kumar ST, Donzelli S, Chiki A, Syed MMK, Lashuel HA (2020) A simple, versatile and robust centrifugation-based filtration protocol for the isolation and quantification of α-synuclein monomers, oligomers and fibrils: towards improving experimental reproducibility in α-synuclein research. J Neurochem 153:103–119. 10.1111/jnc.14955 31925956 PMC7155127

[B21] Jiang C, Haddad GG (1997) Modulation of K+ channels by intracellular ATP in human neocortical neurons. J Neurophysiol 77:93–102. 10.1152/jn.1997.77.1.93 9120601

[B22] Krashia P, Martini A, Nobili A, Aversa D, D'Amelio M, Berretta N, Guatteo E, Mercuri NB (2017) On the properties of identified dopaminergic neurons in the mouse substantia nigra and ventral tegmental area. Eur J Neurosci 45:92–105. 10.1111/ejn.13364 27519559

[B23] Lacey MG, Mercuri NB, North RA (1989) Two cell types in rat substantia nigra zona compacta distinguished by membrane properties and the actions of dopamine and opioids. J Neurosci 9:1233–1241. 2703874 10.1523/JNEUROSCI.09-04-01233.1989 PMC6569880

[B24] Lashuel HA, Overk CR, Oueslati A, Masliah E (2013) The many faces of α synuclein: from structure and toxicity to therapeutic target. Nat Rev Neurosci 14:38–48. 10.1038/nrn3406 23254192 PMC4295774

[B25] Light PE, French RJ (1994) Glibenclamide selectively blocks ATP-sensitive K+ channels reconstituted from skeletal muscle. Eur J Pharmacol 259:219–222. 10.1016/0014-2999(94)90647-5 7982447

[B26] Liss B, Haeckel O, Wildmann J, Miki T, Seino S, Roeper J (2005) K-ATP channels promote the differential degeneration of dopaminergic midbrain neurons. Nat Neurosci 8:1742–1751. 10.1038/nn1570 16299504

[B27] Lu L, Fu DL, Li HQ, Liu AJ, Li JH, Zheng GQ (2014) Diabetes and risk of Parkinson's disease: an updated meta-analysis of case-control studies. PLoS One 9:e85781. 10.1371/journal.pone.0085781 24465703 PMC3897520

[B28] Mahul-Mellier AL, Burtscher J, Maharjan N, Weerens L, Croisier M, Kuttler F, Leleu M, Knott GW, Lashuel HA (2020) The process of Lewy body formation, rather than simply alpha-synuclein fibrillization, is the major driver of neurodegeneration in synucleinopathies. Proc Natl Acad Sci USA 117:4971–4982. 32075919 10.1073/pnas.1913904117 PMC7060668

[B29] Mayer ML, Westbrook GL (1983) A voltage-clamp analysis of inward (anomalous) rectification in mouse spinal sensory ganglion neurones. J Physiol 340:19–45. 10.1113/jphysiol.1983.sp014747 6887047 PMC1199194

[B30] Michel PP, Hirsch EC, Hunot S (2016) Understanding dopaminergic cell death pathways in Parkinson disease. Neuron 90:675–691. 10.1016/j.neuron.2016.03.038 27196972

[B31] Mironov S (2015) α-Synuclein forms non-selective cation channels and stimulates ATP-sensitive potassium channels in hippocampal neurons. J Physiol 593:145–159. 10.1113/jphysiol.2014.280974 25556793 PMC4293060

[B32] Murphy DD, Rueter SM, Trojanowski JQ, Lee VMY (2000) Synucleins are developmentally expressed, and α-synuclein regulates the size of the presynaptic vesicular pool in primary hippocampal neurons. J Neurosci 20:3214–3220. 10.1523/JNEUROSCI.20-09-03214.2000 10777786 PMC6773130

[B33] Neuhoff H, Neu A, Liss B, Roeper J (2002) I(h) channels contribute to the different functional properties of identified dopaminergic subpopulations in the midbrain. J Neurosci 22:1290–1302. 11850457 10.1523/JNEUROSCI.22-04-01290.2002 PMC6757558

[B34] Patel JC, Witkovsky P, Coetzee WA, Rice ME (2011) Subsecond regulation of striatal dopamine release by pre-synaptic KATP channels. J Neurochem 118:721–736. 10.1111/j.1471-4159.2011.07358.x 21689107 PMC3369699

[B35] Polinski NK, Volpicelli-Daley LA, Sortwell CE, Luk KC, Cremades N, Gottler LM, Froula J, Duffy MF, Lee VMY, Martinez TN, Dave KD (2018) Best practices for generating and using alpha-synuclein pre-formed fibrils to model Parkinson's disease in rodents. J Parkinsons Dis 8:303–322. 10.3233/JPD-171248 29400668 PMC6004926

[B36] Richards CD, Shiroyama T, Kitai ST (1997) Electrophysiological and immunocytochemical characterization of GABA and dopamine neurons in the substantia nigra of the rat. Neuroscience 80:545–557. 10.1016/s0306-4522(97)00093-6 9284356

[B37] Schernhammer E, Hansen J, Rugbjerg K, Wermuth L, Ritz B (2011) Diabetes and the risk of developing Parkinson's disease in Denmark. Diabetes Care 34:1102–1108. 10.2337/dc10-1333 21411503 PMC3114482

[B38] Schiemann J, Schlaudraff F, Klose V, Bingmer M, Seino S, Magill P, Zaghloul KA, Schneider G, Liss B, Roeper J (2012) K-ATP channels in dopamine substantia nigra neurons control bursting and novelty-induced exploration. Nat Neurosci 15:1272–1280. 10.1038/nn.3185 22902720 PMC4242970

[B39] Schwanstecher M, Sieverding C, Dörschner H, Gross I, Aguilar-Bryan L, Schwanstecher C, Bryan J (1998) Potassium channel openers require ATP to bind to and act through sulfonylurea receptors. EMBO J 17:5529–5535. 10.1093/emboj/17.19.5529 9755153 PMC1170881

[B40] Seino S (1999) ATP-sensitive potassium channels: a model of heteromultimeric potassium channel/receptor assemblies. Annu Rev Physiol 61:337–362. 10.1146/annurev.physiol.61.1.337 10099692

[B41] Spillantini MG, Schmidt ML, Lee VMY, Trojanowski JQ, Jakes R, Goedert M (1997) α-Synuclein in Lewy bodies. Nature 388:839–840. 10.1038/42166 9278044

[B42] Stanford IM, Lacey MG (1995) Regulation of a potassium conductance in rat midbrain dopamine neurons by intracellular adenosine triphosphate (ATP) and the sulfonylureas tolbutamide and glibenclamide. J Neurosci 15:4651–4657. 7790930 10.1523/JNEUROSCI.15-06-04651.1995 PMC6577743

[B43] Thakur P, Luk K, Roeper J (2019) Selective K-ATP channel-dependent loss of pacemaking in vulnerable nigrostriatal dopamine neurons by α-synuclein aggregates. bioRxiv 842344 doi: 10.1101/842344.

[B44] Wahlqvist M, Lee MS, Hsu CC, Chuang SY, Lee J, Tsai HN (2012) Metformin-inclusive sulfonylurea therapy reduces the risk of Parkinson's disease occurring with Type 2 diabetes in a Taiwanese population cohort. Parkinsonism Relat Disord 18:753–758. 10.1016/j.parkreldis.2012.03.010 22498320

[B45] Winner B, Jappelli R, Maji SK, Desplats PA, Boyer L, Aigner S, Hetzer C, Loher T, Vilar M, Campioni S, Tzitzilonis C, Soragni A, Jessberger S, Mira H, Consiglio A, Pham E, Masliah E, Gage FH, Riek R (2011) In vivo demonstration that α-synuclein oligomers are toxic. Proc Natl Acad Sci USA 108:4194–4199. 10.1073/pnas.1100976108 21325059 PMC3053976

[B46] Zhang Q, Li C, Zhang T, Ge Y, Han X, Sun S, Ding J, Lu M, Hu G (2018) Deletion of Kir6.2/SUR1 potassium channels rescues diminishing of DA neurons via decreasing iron accumulation in PD. Mol Cell Neurosci 92:164–176. 10.1016/j.mcn.2018.08.006 30171894

